# Long-Term PM_2.5_ Exposure and Clinical Skin Aging: A Systematic Review and Meta-Analysis of Pigmentary and Wrinkle Outcomes

**DOI:** 10.3390/life16010061

**Published:** 2025-12-30

**Authors:** Jeng-Wei Tjiu, Chia-Fang Lu

**Affiliations:** 1 Department of Dermatology, National Taiwan University Hospital, National Taiwan University College of Medicine, Taipei 100, Taiwan; 2 Grace Smile Dental Clinic, Taipei 100, Taiwan

**Keywords:** PM_2.5_, air pollution, skin aging, photoaging, lentigines, pigment spots, wrinkles, VISIA imaging, extrinsic aging, environmental dermatology

## Abstract

Background: Fine particulate matter (PM_2.5_) is an established systemic toxicant, yet its association with clinical skin aging remains incompletely characterized. Although pigmentary changes and wrinkles are commonly attributed to ultraviolet exposure, experimental and epidemiologic evidence suggests that long-term PM_2.5_ exposure may contribute to extrinsic skin aging through oxidative, inflammatory, and aryl hydrocarbon receptor-mediated pathways. However, human studies specifically quantifying PM_2.5_ exposure in relation to validated skin aging outcomes are sparse, and no prior meta-analysis has systematically synthesized this evidence. Objective: To conduct a systematic review and meta-analysis of epidemiologic studies reporting measured or modeled long-term PM_2.5_ exposure and extractable quantitative associations with clinical skin aging outcomes. Methods: We performed a comprehensive PRISMA 2020-guided search of PubMed, Embase, Web of Science, and Scopus (inception to 18 November 2025). Eligible studies included human participants, quantified long-term PM_2.5_ exposure, validated clinical or imaging-based skin aging outcomes, and extractable effect estimates. Ratio-type effect measures (arithmetic mean ratios, geometric mean ratios, and odds ratios) were transformed to the natural-log scale, standardized to a common exposure contrast of per 10 µg/m^3^ PM_2.5_, and synthesized as generic relative association metrics. Random-effects models with DerSimonian–Laird estimation and Hartung–Knapp adjustment were applied for pigmentary outcomes. VISIA imaging β-coefficients were synthesized narratively. Results: Four epidemiologic cohorts met predefined eligibility criteria. From these, we extracted seven PM_2.5_-specific pigmentary effect estimates, one clinically assessed wrinkle estimate, and two VISIA imaging outcomes. The pooled relative association for pigmentary aging corresponded to a ratio of 1.11 per 10 µg/m^3^ PM_2.5_ (95% CI, 0.82–1.50), indicating a directionally positive but statistically imprecise association compatible with both increased and unchanged pigmentary aging. All individual pigmentary estimates were directionally positive. A single cohort reported a 3.2% increase in wrinkle severity per 10 µg/m^3^ PM_2.5_ (ratio 1.032). VISIA imaging showed significant worsening of brown spot severity (+9.5 percentile per 10 µg/m^3^), while wrinkle percentiles showed a non-significant change. Conclusions: Based on a comprehensive PRISMA-guided search, the available epidemiologic evidence suggests a consistent directionally positive association between long-term PM_2.5_ exposure and pigmentary skin aging outcomes, with limited and uncertain evidence for wrinkle-related phenotypes. The current evidence base remains small, heterogeneous, and of low certainty. Accordingly, these findings should be interpreted as hypothesis-generating and underscore the need for larger, longitudinal, and methodologically harmonized studies. (Registration: PROSPERO CRD420251231462)

## 1. Introduction

Extrinsic skin aging is driven by chronic environmental exposures, including ultraviolet (UV) radiation, cigarette smoke, and atmospheric pollutants [1,2]. Among these, fine particulate matter (PM_2.5_) has emerged as a major global health concern because of its deep respiratory penetration, systemic distribution, and well-established associations with cardiovascular, pulmonary, and metabolic aging [3,4,5]. While the systemic health effects of PM_2.5_ are extensively documented, its contribution to clinically observable skin aging remains comparatively underexplored [6,7].

Mechanistic evidence strongly supports a potential role for PM_2.5_ in cutaneous aging processes [6,7,8]. PM_2.5_ carries adsorbed polycyclic aromatic hydrocarbons, heavy metals, and organic carbon species that generate reactive oxygen species (ROS) [9,10,11], activate the aryl hydrocarbon receptor (AhR) [12,13], and induce matrix metalloproteinases (MMP-1 and MMP-3) [14], enzymes implicated in collagen degradation and wrinkle formation. In parallel, PM_2.5_ exposure has been shown to upregulate melanogenic pathways [12,13,14,15], disrupt epidermal barrier function, and promote chronic low-grade inflammation [10], contributing to lentigines and pigmentary changes. Experimental and epidemiologic studies further suggest synergistic interactions between PM_2.5_ and ultraviolet radiation, whereby particulate pollutants may potentiate UV-induced pigmentation and lentigo formation [6,16].

Despite these biologically plausible mechanisms, epidemiologic evidence linking long-term PM_2.5_ exposure to clinical skin aging remains limited. Available studies are sparse, geographically heterogeneous, and inconsistent in outcome definitions and phenotyping approaches [6,7]. To date, only a small number of human studies have quantitatively evaluated measured or modeled long-term PM_2.5_ exposure in relation to validated clinical skin aging outcomes, such as solar lentigines, pigment spots, wrinkles, or VISIA-derived photoaging metrics [16,17,18,19]. Importantly, no prior systematic review has explicitly isolated PM_2.5_ exposure, which is distinct from broader or composite air pollution indices, and quantitatively synthesized its association with clinical skin aging phenotypes [2,6,7].

Given the global relevance of PM_2.5_ exposure and growing interest in pollution-related skin damage, a rigorous quantitative synthesis of the available epidemiologic evidence is warranted [2,3,4,7]. Accordingly, we conducted a PRISMA-guided systematic review and meta-analysis of epidemiologic studies reporting (1) long-term PM_2.5_ exposure assessed via ambient monitoring, land-use regression, or indoor modeling approaches, and (2) extractable quantitative associations with clinical skin aging outcomes [20,21,22]. By harmonizing heterogeneous effect metrics to a common exposure contrast of per 10 µg/m^3^ PM_2.5_ and applying random-effects models with Hartung–Knapp adjustment, we aimed to summarize the direction, magnitude, and uncertainty of reported associations [23,24,25].

This review synthesizes the currently identifiable epidemiologic evidence on long-term PM_2.5_ exposure and clinical skin aging based on a comprehensive PRISMA-guided search. Given the limited number of eligible cohorts and the anticipated imprecision of pooled estimates, this work is intended primarily as a hypothesis-generating synthesis rather than a definitive assessment of clinical or public health impact.

## 2. Materials and Methods

This systematic review and meta-analysis was conducted and reported in full accordance with the PRISMA 2020 guidelines [20,21] and prospectively registered in PROSPERO (CRD420251231462) [26]. A comprehensive literature search was performed in PubMed, Embase, Web of Science, and Scopus [22,27,28] using a combination of controlled vocabulary (MeSH and Emtree terms) and free-text keywords (Supplementary Table S1) [29,30]. The completed PRISMA 2020 checklist is provided in Supplementary Table S2, and the PRISMA Abstract checklist is presented in Supplementary Table S3. In addition to database searches, we manually screened reference lists, tracked citations forward and backward [26,31], reviewed the relevant gray literature [32], and screened publications citing the four key cohorts (Ding 2017; Peng 2017; Hüls 2019; Huang 2022) [16,17,18,19].

### 2.1. Eligibility Criteria

Studies were selected according to predefined eligibility criteria based on the Population–Exposure–Outcome–Study Design (PEOS) framework [33,34]. The objective of this review was to identify epidemiologic investigations that quantified long-term exposure to fine particulate matter (PM_2.5_) in relation to clinically assessed skin aging outcomes [5,6,7].

#### 2.1.1. Inclusion Criteria

To be eligible for inclusion, studies were required to meet criteria across population, exposure, outcomes, effect measures, and study design [22,35]. Eligible populations included human participants of any age, sex, or ethnicity drawn from clinical, community, or population-based settings [22]. Long-term PM_2.5_ exposure was required to be measured or modeled as annual or multi-year mass concentrations using governmental monitoring networks, land-use regression-derived estimates, satellite-based PM_2.5_ integrated with chemical transport models, indoor PM_2.5_ measurements or calibrated indoor modeling approaches (e.g., Ding 2017 [17]), or regional or district-level contrasts with quantifiable concentration differences [16,17,18,36,37,38,39]. A fundamental requirement was that PM_2.5_ exposure be expressed as a mass concentration in micrograms per cubic meter (µg/m^3^) or be convertible to this unit [3].

Eligible studies were required to assess at least one validated clinical or imaging-based skin aging phenotype, including lentigines, pigment spots, wrinkles, SCINEXA subscores, or VISIA metrics [6,19,40,41]. Only validated, dermatologist-assessed, or instrument-based outcome measures were accepted [6,19,40,41]. To ensure analytic compatibility, studies were required to report extractable quantitative associations—odds ratios (ORs), risk ratios (RRs), arithmetic mean ratios (AMRs), geometric mean ratios (GMRs), or β-coefficients—with sufficient information to permit variance estimation [35,42,43,44,45].

Acceptable study designs included cross-sectional studies, cohort studies, case–control studies, and population-based surveys with validated exposure and outcome assessments [22,35,44,46]. Randomized or experimental designs were not required given the observational nature of environmental epidemiology [44,46].

#### 2.1.2. Exclusion Criteria

Studies were excluded if exposure assessment was not PM_2.5_-specific—for example, if they reported only PM_10_, nitrogen dioxide, ozone, or composite air pollution indices without distinct PM_2.5_ mass measurements [4,47,48]. Studies relying solely on proxy exposures, such as household solid-fuel use, biomass indicators, elemental carbon, or soot absorbance without PM_2.5_ mass concentration data, were also excluded [48,49,50]. Studies were ineligible if their outcomes were unrelated to skin aging, including investigations focused exclusively on erythema, barrier function, inflammation, dermatitis, or general skin symptoms without age-related endpoints [7,14]. Short-term or acute PM_2.5_ exposure studies with exposure windows shorter than 14 days were excluded [8,51], as were studies that provided no extractable effect estimates or insufficient quantitative information to permit effect size transformation [22,35,43,52]. Non-human studies, including animal experiments, in vitro analyses, cosmetic product trials, and mechanistic laboratory investigations, were also excluded.

#### 2.1.3. Study Selection

Study selection was conducted in two stages [20,21,22]. During initial title and abstract screening, all retrieved records were assessed for relevance to long-term PM_2.5_ exposure and clinical skin aging outcomes [20,21,22]. Full texts of potentially eligible studies were then reviewed to verify that PM_2.5_ exposure had been quantified as a mass concentration and that outcomes represented validated indicators of skin aging with extractable effect estimates [1,22,39,45]. Studies were excluded at this stage if they lacked PM_2.5_-specific exposure data, assessed outcomes unrelated to skin aging, or did not provide sufficient variance information for quantitative synthesis [22,39,45,46]. All PM_2.5_ concentrations are reported in micrograms per cubic meter (µg/m^3^).

### 2.2. Data Extraction and Quality Assessment

#### 2.2.1. Data Extraction

A standardized data extraction form was developed a priori to ensure consistent capture of all relevant study characteristics [20,22,39]. Two reviewers independently extracted data from each eligible article and Supplementary Materials [20,22,39]. Extracted study-level information included study identifiers, cohort characteristics, study design, sample size, and inclusion and exclusion criteria [22,33].

For PM_2.5_ exposure assessment, reviewers recorded the exposure estimation approach, including ambient monitoring, land-use regression (e.g., the SALIA cohort), calibrated indoor PM_2.5_ models (e.g., Ding 2017), and regional or district-level contrasts (e.g., Peng 2017) [17,18,34,35,36]. All PM_2.5_ concentrations are reported in micrograms per cubic meter (µg/m^3^). Additional exposure details, including exposure windows and distributional metrics (mean, standard deviation, and interquartile range [IQR]), were extracted when available [3].

Skin aging outcomes were extracted by phenotype category—lentigines, pigment spots, SCINEXA subscores (Score of Intrinsic and Extrinsic Skin Aging), VISIA^®^ computerized facial imaging outcomes (percentile scores), and wrinkle severity—and by assessment method (dermatologist rating, SCINEXA scoring, VISIA imaging, or ordinal grading) [6,19,38,49]. All effect estimates relevant to PM_2.5_ exposure (odds ratios [ORs], risk ratios [RRs], arithmetic mean ratios [AMRs], geometric mean ratios [GMRs], and β-coefficients), together with their 95% confidence intervals and corresponding exposure contrasts (per IQR, per µg/m^3^, or high–low contrasts), were extracted [3,20,22,39].

For studies reporting outcomes at multiple anatomical sites (e.g., Ding 2017; Hüls 2019), effect estimates were extracted separately where phenotypes differed [16,17]. When effect sizes were reported only in graphical form, numerical values were obtained from Supplementary Materials in accordance with standard environmental epidemiology practice [53,54]. Information on covariate adjustment—including age, sex, skin type, ultraviolet exposure, smoking status, body mass index (BMI), socioeconomic status, and skincare-related variables—was extracted and independently verified [2]. Exposure contrasts were recalculated or confirmed using reported distributions (e.g., PM_2.5_ IQR = 1.9 µg/m^3^ in Hüls 2019) [3,16]. All extracted values were cross-validated against Supplementary Materials and original reports (e.g., Ding 2017; Hüls 2019) [3,16,17].

#### 2.2.2. Quality Assessment

Risk of bias for each study was assessed independently by two reviewers using a modified Newcastle–Ottawa Scale (NOS) adapted for environmental epidemiology [22,55,56,57]. The assessment encompassed five primary domains. In the Selection domain, reviewers evaluated the representativeness of the study population, clarity of inclusion criteria, and participation rates [22,55,56]. In the exposure assessment domain, emphasis was placed on the precision and validity of PM_2.5_ measurement or modeling, including spatial and temporal resolution and the extent of model validation, with particular attention to potential exposure misclassification [34,36,37].

Outcome Assessment was evaluated based on the validity and reproducibility of skin aging measurements (e.g., SCINEXA scoring, lentigines grading, VISIA imaging) and whether Outcome Assessment was blinded, if applicable [2,6,38,49]. The Confounding domain assessed whether analyses adjusted for key covariates known to influence skin aging and PM_2.5_ exposure, including age, ultraviolet radiation exposure, smoking, skin type, socioeconomic status, and BMI, and whether UV-related behavioral variables were incorporated, given their relevance to pigmentary outcomes [12,19]. Analytical Quality was evaluated by reviewing the appropriateness of statistical models, the completeness of variance reporting, and the transparency of exposure scaling procedures [22,40,46,57].

Each study was assigned an overall risk of bias rating (low, moderate, or high) based on cumulative NOS scoring [22,55]. Full item-level assessments for all included studies are presented in Section 3.

### 2.3. Statistical Analysis

All statistical analyses were conducted in accordance with established best practices in environmental epidemiology and small-study meta-analysis [22,25,37,58]. Because the included studies reported heterogeneous effect measures—including arithmetic mean ratios (AMRs), geometric mean ratios (GMRs), odds ratios (ORs), and β-coefficients—and used differing PM_2.5_ exposure contrasts, we implemented a structured harmonization framework to enable comparability across datasets [3,34,40,46,59].

Because fewer than ten studies contributed to quantitative syntheses, several standard meta-analytic procedures were not performed. Specifically, formal subgroup meta-analyses, meta-regression, and statistical tests for publication bias (e.g., Egger’s or Begg’s tests) were not conducted, as these methods are unreliable and potentially misleading in sparse-evidence settings. Where relevant, descriptive or qualitative assessments were used instead. This constraint applies to all quantitative analyses presented in this manuscript.

#### 2.3.1. Effect Size Harmonization

For ratio-type outcomes (AMRs, GMRs, and ORs), effect estimates were transformed to the natural-log scale and standardized to represent the association per 10 µg/m^3^ increment in PM_2.5_ exposure [22,34,46,59]. Although AMRs, GMRs, and ORs arise from different outcome models, all represent multiplicative contrasts and can be expressed on a common log scale. These ratio-type measures were harmonized and pooled solely as generic relative association metrics to summarize the direction and relative magnitude of associations between long-term PM_2.5_ exposure and pigmentary skin aging outcomes.

The pooled log-ratio was not interpreted as a unified causal estimand, an absolute risk, a probability, or an interchangeable clinical severity measure. Rather, it represents a descriptive summary of relative associations on the log scale, consistent with established practice in environmental epidemiology when evidence is sparse and outcomes are directionally comparable. No conversion of AMRs or GMRs into ORs was undertaken; all ratio-type measures were retained in their original form and harmonized only through log-scale rescaling to a common exposure contrast.

Log transformation was performed using θ = ln(effect). Standard errors were derived from reported 95% confidence intervals as SE = [ln(CI_upper) − ln(CI_lower)]/(2 × 1.96) [22,46,59]. When effect estimates were reported per interquartile range (IQR) or per high–low exposure contrast, log-scale estimates were rescaled to a per–10 µg/m^3^ contrast using θ_10_ = θ × (10/Contrast) and SE_10_ = SE × (10/Contrast) [3,34,58,60], where Contrast corresponded to the reported IQR (e.g., 28.93 µg/m^3^ in Ding 2017, 1.9 µg/m^3^ in Hüls 2019) or a categorical exposure difference (e.g., 104.57 µg/m^3^ in Peng 2017) [16,17,18].

These harmonized estimates formed the analytic basis for the quantitative synthesis of pigmentary outcomes and the single extractable wrinkle outcome [22,46]. VISIA-based aging outcomes were reported as continuous β-coefficients rather than ratios and therefore excluded from quantitative pooling to avoid cross-estimand mixing. VISIA β-coefficients were linearly rescaled from per 1 µg/m^3^ to per 10 µg/m^3^ increments and synthesized narratively [54,61].

In this manuscript, the term severity is used descriptively to denote higher values on the outcome scales employed by individual studies (e.g., greater lesion counts, higher ordinal grades, or worse composite scores). Because outcomes were measured on different scales, pooled estimates represent relative associations on the log scale rather than a single unified or directly comparable clinical severity metric.

#### 2.3.2. Meta-Analytic Model

Given anticipated heterogeneity arising from differences in PM_2.5_ exposure assessment methods, skin aging phenotype definitions, and regional background pollution, a random-effects meta-analytic model was applied [22,58,62]. Between-study variance (τ^2^) was estimated using the DerSimonian–Laird method, which is commonly used in environmental exposure meta-analyses [23,46,62]. Because the number of studies contributing to pooled estimates was small (k < 10), the Hartung–Knapp–Sidik–Jonkman adjustment was applied to compute conservative and robust confidence intervals [22,24,63,64].

Pooled estimates were calculated using inverse-variance weighting, with weights defined as w_i_ = 1/(SE_i_^2^ + τ^2^). Uncertainty was quantified using Cochran’s Q statistic and a t-distribution with degrees of freedom equal to k − 1 [22,24,62]. This approach provides greater inferential stability than conventional Wald-type confidence intervals in small-sample meta-analyses [24,25,63,64].

To assess robustness under sparse-study conditions, sensitivity analyses were performed using alternative between-study variance estimators. Specifically, random-effects models were re-estimated using restricted maximum likelihood (REML) and Paule–Mandel τ^2^ estimators, each combined with Hartung–Knapp–Sidik–Jonkman confidence intervals.

#### 2.3.3. Heterogeneity and Inconsistency

Between-study heterogeneity was assessed using Cochran’s Q statistic, between-study variance (τ^2^), and the I^2^ statistic, which reflects the proportion of total variability attributable to true heterogeneity rather than sampling error [22,23,62]. Given known variability in PM_2.5_ exposure modeling approaches and dermatologic phenotyping methods, moderate to high I^2^ values were anticipated and interpreted accordingly [2,6,34,58].

#### 2.3.4. Prediction Intervals

Prediction intervals were calculated to estimate the range of true effects that may be observed in future studies. Given the small number of contributing studies, 95% prediction intervals were computed using a t-distribution with degrees of freedom equal to k − 2, rather than a normal approximation. Specifically, prediction intervals were calculated as PI = θ^ ± t_0.975_, (k − 2) × √(τ^2^ + Var(θ^)), where θ^ denotes the pooled log-ratio estimate and τ^2^ represents the between-study variance [22,25,40,62]. Prediction intervals incorporate both within- and between-study uncertainty and are therefore recommended for heterogeneous observational evidence, particularly in small-sample settings [22,25,46,65].

#### 2.3.5. Sensitivity Analyses

Sensitivity analyses were conducted to evaluate the influence of individual studies on pooled estimates [25,40,46]. Leave-one-out analyses were performed to assess robustness [25,46], and a targeted influence assessment was conducted for Hüls 2019 because its narrow PM_2.5_ IQR and relatively large variance could disproportionately affect pooled results [25,34,40]. Additional comparisons examined estimates derived from indoor PM_2.5_ exposure models (Ding 2017) versus those derived from ambient PM_2.5_ measurements (Peng 2017; Hüls 2019; Huang 2022) [16,17,18,19]. Formal statistical tests for small-study publication bias were not performed, as these methods require at least ten studies to yield reliable results [22,44,66,67].

#### 2.3.6. Software

All statistical analyses were performed in MATLAB R2023a using custom scripts developed for effect size harmonization, variance derivation from confidence intervals, random-effects meta-analysis with Hartung–Knapp–Sidik–Jonkman adjustment, prediction interval calculation, and sensitivity analyses. The complete MATLAB scripts are provided in Supplementary File S1 to ensure reproducibility [22,25,68,69]. These scripts were also used to generate all meta-analytic tables and publication-quality figures [70].

### 2.4. Risk of Bias Assessment

The internal validity of each included study was evaluated independently by two reviewers using a modified Newcastle–Ottawa Scale (NOS) tailored for environmental epidemiology and dermatology research [22,55,56,57]. This adapted instrument assessed methodological quality across five domains—Selection, Exposure Assessment, Outcome Assessment, Control of Confounding, and Analytical Quality—reflecting the principal sources of bias relevant to PM_2.5_ exposure and clinical skin aging research [22,34,38,57,58]. Any discrepancies between reviewers were resolved through discussion and consensus [22,56]. Full item-level risk of bias assessments are presented in Section 3.

#### 2.4.1. Selection Domain

The Selection domain evaluated the representativeness of the study sample, the clarity of inclusion and exclusion criteria, the adequacy of recruitment methods, and the potential for selection bias arising from differential participation [22,33,50,71]. Studies based on community or population-level samples, such as the SALIA cohort and the Taizhou population, were judged to have a lower risk of selection bias [16,17,22]. In contrast, dermatology-clinic-based samples were categorized as moderate risk because of potential enrichment for individuals with pre-existing skin concerns [72,73,74].

#### 2.4.2. Exposure Assessment Domain

Because PM_2.5_ exposure estimation varied substantially across cohorts, the Exposure Assessment domain focused on the precision and validity of exposure measurement methods, including governmental monitoring data, land-use regression (LUR) modeling, and calibrated indoor PM_2.5_ measurement systems [17,34,35,36,37]. Spatial resolution (e.g., grid-level predictions versus district-level assignment), temporal averaging (annual or multi-year means), and susceptibility to exposure misclassification were also considered [58,75,76,77]. Studies employing validated LUR models, as in Hüls 2019, or calibrated indoor PM_2.5_ models, as in Ding 2017, were rated as low risk [16,17,34,35,36]. In contrast, studies relying on broader district-level contrasts, such as Peng 2017, were rated as moderate risk because of increased uncertainty in exposure assignment [18,37,58,76].

#### 2.4.3. Outcome Assessment Domain

The Outcome Assessment domain examined the validity and reproducibility of skin aging phenotypes, use of objective or instrument-based measurements, application of standardized or blinded dermatologist ratings when applicable, and consistency in anatomical site selection [6,16,17,19,38,49]. VISIA-based imaging outcomes (Huang 2022) and SCINEXA-derived lentigines scoring (Ding 2017; Hüls 2019) were rated as low risk due to their objective and reproducible assessment methods [16,17,19,38]. In contrast, studies relying on non-blinded ordinal grading or mixed anatomical site assessments were judged to have a moderate risk of bias [49,78].

#### 2.4.4. Confounding Domain

This domain evaluated the adequacy of adjustment for key confounders known to influence both skin aging and PM_2.5_ exposure, including chronological age, sex, Fitzpatrick skin type, ultraviolet exposure patterns, smoking and secondhand smoke exposure, body mass index (BMI), socioeconomic indicators, and skincare or sunscreen use when available [2,6,14,79,80]. Cohorts with comprehensive multivariable adjustment—most notably, Ding 2017 and Hüls 2019—were judged to have a low risk of residual confounding. Studies with partial or incomplete adjustment, such as Peng 2017 and Huang 2022, were assessed as having a moderate risk of residual confounding [16,17,18,19].

#### 2.4.5. Analytical Quality Domain

The Analytical Quality domain assessed the transparency of PM_2.5_ exposure scaling and contrast definitions (e.g., interquartile range contrasts, high–low comparisons, or continuous increments), the reporting of confidence intervals and variance estimates, the appropriateness of statistical models, and clarity in the handling of multiple anatomical sites [3,22,34,46,58,59]. Studies lacking explicit variance estimates, effect size scaling details, or sufficient methodological description were considered at higher risk in this domain [22,40,46,58,59]. In contrast, studies such as Hüls 2019, which provided complete supplementary numeric data and detailed ordinal regression outputs, were rated as low risk [16].

#### 2.4.6. Overall Risk of Bias Judgment

Each study was classified as having a low, moderate, or high overall risk of bias based on cumulative domain-level ratings [22,55,56,57]. Ding 2017 and Hüls 2019 were judged to be at low overall risk of bias [16,17], whereas Peng 2017 and Huang 2022 were assessed as having moderate overall risk [18,19]. No study was classified as high risk after excluding studies that relied on proxy-based exposure measures, such as household solid-fuel use [42,51]. Overall, the available evidence base demonstrated reasonable methodological quality, although conclusions remain constrained by the small number of PM_2.5_-specific skin aging studies worldwide [22,55,58].

### 2.5. Data Synthesis

#### 2.5.1. Preparation of Ratio-Type Effect Sizes

Given heterogeneity in exposure contrasts and effect size metrics, all ratio-type measures—including odds ratios, risk ratios, arithmetic mean ratios, and geometric mean ratios—were first log-transformed to stabilize variance [22,46,59]. Each study’s effect estimate was then harmonized to a common exposure contrast of per 10 µg/m^3^ PM_2.5_ using study-specific interquartile ranges or reported high–low concentration differences [3,22,34,58]. Variances were derived from published 95% confidence intervals. Effect sizes were pooled only when studies evaluated the same clinical construct, specifically pigmentary aging or wrinkle severity [22,40,46,59].

Through this harmonization process, seven PM_2.5_-specific pigmentary effect sizes from four independent cohorts and one PM_2.5_-specific wrinkle effect size were obtained [16,17,18,34]. VISIA-derived outcomes were reported as continuous β-coefficients and therefore incompatible with ratio-type measures; these outcomes were synthesized narratively, as described in Section 2.5.3 [54,61].

Accordingly, all pooled estimates are interpreted as relative associations on the log scale per 10 µg/m^3^ PM_2.5_ and should not be construed as absolute or directly comparable clinical effect sizes across differing outcome models. In this manuscript, the term *severity* is used descriptively to denote higher values on the outcome scales employed in individual studies (e.g., greater lesion counts, higher ordinal grades, or worse composite scores). Because outcomes were measured on different scales, pooled estimates represent relative associations rather than a single unified or directly comparable clinical severity metric.

#### 2.5.2. Quantitative Synthesis (Meta-Analysis)

Pigmentary aging outcomes (k = 7) were synthesized quantitatively because the included studies evaluated closely related phenotypes, such as lentigines counts, pigment spot severity, and SCINEXA pigment subscores [45,46,59]. A random-effects meta-analytic model was applied using the DerSimonian–Laird estimator to quantify between-study variance (τ^2^) [23,58,62], with Hartung–Knapp adjustment used to generate robust confidence intervals appropriate for meta-analyses with fewer than ten studies [24,25,63].

Effect sizes were pooled on the log scale and subsequently back-transformed to express the pooled association as a relative change per 10 µg/m^3^ PM_2.5_ [22,58,62]. For wrinkle outcomes, only a single study (Ding 2017) provided sufficient data to extract a PM_2.5_-specific effect estimate; accordingly, wrinkle findings were summarized descriptively and presented in forest plot format for visual consistency without formal pooling [17,22,61].

#### 2.5.3. Narrative Synthesis for VISIA Imaging Outcomes

VISIA imaging outcomes required separate treatment because they were reported as β-coefficients rather than ratio-type measures, precluding integration with the quantitative meta-analysis [22,54,61]. These β-coefficients were recalibrated to represent a per 10 µg/m^3^ PM_2.5_ exposure increment [19,22,61]. Results were synthesized narratively, and a dedicated VISIA imaging figure was produced to present effect estimates with corresponding confidence intervals [22]. This approach follows Cochrane guidance for synthesizing heterogeneous effect measures that cannot be pooled statistically [22,54].

#### 2.5.4. Heterogeneity and Prediction Intervals

Heterogeneity across pigmentary aging studies was assessed using Cochran’s Q statistic, the I^2^ statistic, and the between-study variance (τ^2^) [22,23]. Because differences in exposure assessment methods, such as indoor modeling, ambient monitoring, and land-use regression, were expected to contribute to between-study variability, 95% prediction intervals were calculated to estimate the plausible range of true effects that may be observed in future populations or study settings [25,45,62,65].

#### 2.5.5. Sensitivity Analyses

Sensitivity analyses included leave-one-out procedures to evaluate the influence of individual studies on the pooled estimate [45,46,81]. A targeted influence assessment was also conducted for Hüls 2019 because its relatively narrow PM_2.5_ interquartile range could affect comparability with other cohorts [16,34,75]. Exploratory comparisons of indoor versus ambient PM_2.5_ exposure sources were performed [17,18,19]. Formal statistical tests for publication bias were not undertaken, as methods for detecting funnel plot asymmetry are unreliable when fewer than ten studies are available [22,44,66,67].

#### 2.5.6. Data Presentation

Results are presented using forest plots for ratio-type outcomes, a dedicated VISIA imaging plot, study-level summary tables, and comprehensive supplementary extraction tables in Section 3 [21,22,46,54]. All synthesis procedures adhered to established best practices for environmental epidemiology, particularly for small and heterogeneous exposure–outcome datasets [25,34,40,58].

### 2.6. Subgroup and Sensitivity Analysis

Given the limited number of epidemiological studies evaluating PM_2.5_-specific associations with clinical skin aging—four independent cohorts contributing seven pigmentary effect sizes—subgroup analyses were conducted descriptively, while sensitivity analyses followed established best practices for small-sample environmental meta-analyses [22,25,40,46,58,81,82]. Because fewer than ten studies contributed to quantitative syntheses, formal subgroup meta-analyses, meta-regression, and statistical tests for publication bias (e.g., Egger’s or Begg’s tests) were not performed, as these approaches are unreliable and potentially misleading in sparse-evidence settings. Where appropriate, descriptive or qualitative comparisons were used instead. This methodological constraint applies to all analyses.

#### 2.6.1. Subgroup Analysis

Formal subgroup meta-analysis generally requires adequate numbers of studies, with typically at least two effect sizes per subgroup [22,41,45,82]. Given the very small evidence base for PM_2.5_ and clinical skin aging, subgroup evaluations were therefore performed descriptively rather than quantitatively [22,41,45].

First, exposure assessment methods were compared qualitatively across studies using calibrated indoor PM_2.5_ models (Ding 2017), ambient monitoring-based estimates (Peng 2017), land-use regression-derived exposures (Hüls 2019), and residential ambient PM_2.5_ assignments (Huang 2022) [16,17,18,19,34,58]. Differences in effect size magnitude across these exposure approaches were considered when interpreting pooled estimates, although no formal subgroup models were estimated because of insufficient study numbers within each category [22,41,45,82].

Second, geographic variation was summarized descriptively. Included studies originated from East Asia (China and Taiwan) and Europe (Germany; SALIA cohort) [6]. These regional comparisons were used to contextualize heterogeneity but did not permit estimation of region-specific pooled effects [22,41,45].

Third, phenotypic outcome types and anatomical sites were examined descriptively to assess whether PM_2.5_ associations appeared stronger for particular phenotypes or facial regions [19,38,49,78]. Because most phenotype- or site-specific groupings contained only one or two effect sizes, no formal subgroup pooling was conducted [22,41,45,82].

#### 2.6.2. Sensitivity Analyses

Because random-effects meta-analyses with small numbers of studies may be disproportionately influenced by individual observations, several prespecified sensitivity analyses were conducted to evaluate the robustness of pooled estimates [25,45,46,81].

Leave-one-out analyses were performed by sequentially omitting each pigmentary effect size to assess whether any single study materially altered the pooled association [45,46,81]. Particular attention was paid to studies with greater imprecision, especially Hüls (2019), which used a relatively small PM_2.5_ interquartile range and yielded wider confidence intervals [16,17,34,75].

To further assess the influence of high-variance or methodologically distinct studies, additional models were estimated excluding the SALIA effect sizes from Hüls (2019) and, separately, excluding the Ding (2017) cohort [16,17,25,34,75]. These analyses evaluated whether pooled estimates were sensitive to inclusion of studies with either the narrowest exposure contrasts or the greatest anatomical site heterogeneity [17,18,34,75].

Sensitivity to exposure source heterogeneity was also examined by comparing results derived from indoor PM_2.5_ exposure models (Ding 2017), ambient monitoring-based exposures (Peng 2017; Huang 2022), and land-use regression-derived exposures (Hüls 2019) [17,18,19,34,58]. Additional phenotype-restricted analyses compared lentigines-only models, with models restricted to SCINEXA or pigment spot outcomes [6,38,49]. These comparisons were used to explore whether observed heterogeneity was more likely attributable to differences in pigmentation measurement rather than exposure estimation [6,38,49,78].

Because several cohorts contributed multiple anatomical-site-specific effect estimates, prespecified sensitivity analyses were also conducted to address potential unit of analysis bias arising from within-study non-independence. First, a restricted analysis retained only one pigmentary outcome per cohort, prioritizing the primary facial site reported by each study. Second, for cohorts reporting multiple pigmentary outcomes, within-study effect estimates were averaged on the natural-log scale to generate a single study-level estimate, with variance approximated using inverse-variance weighting. These analyses evaluated the robustness of conclusions to alternative handling of within-study clustering.

#### 2.6.3. Publication Bias Assessment

Formal statistical tests for publication bias, including Egger’s regression and Begg’s test, were not performed because such methods require a minimum of ten studies to yield reliable results [22,44,66,67,83]. An exploratory funnel plot is provided in Section 3; however, interpretation is limited and should be approached with caution [44,66,83,84].

#### 2.6.4. Summary of Sensitivity Findings

Across all sensitivity analyses—including leave-one-out models, exclusion of high-variance studies, exposure–source comparisons, phenotype-restricted analyses, and alternative handling of within-study clustering—the direction of association between long-term PM_2.5_ exposure and pigmentary skin aging remained consistently positive [25,45,46,81]. These convergent findings suggest that the observed pooled association is robust to substantial variability in exposure modeling approaches, phenotype definitions, and study-level precision, although overall interpretation remains constrained by the limited size of the evidence base [25,34,45,58,75].

### 2.7. Certainty of Evidence (GRADE)

The overall certainty of evidence for each outcome domain was evaluated using the GRADE (Grading of Recommendations, Assessment, Development, and Evaluations) framework, adapted for observational environmental epidemiology [53,85,86,87]. Because all included studies were observational, the initial certainty rating for each outcome began at *low* and was subsequently downgraded or, where appropriate, considered for upgrading based on predefined criteria, including risk of bias, inconsistency, indirectness, imprecision, and publication bias [53,85,86]. Potential upgrading was considered for large effect sizes, evidence of a dose–response relationship, or strong mechanistic plausibility [53,85,87]. Certainty ratings were applied separately to pigmentary aging, wrinkle severity, and VISIA-derived imaging outcomes [86,87].

#### 2.7.1. Pigmentary Skin Aging (Overall Certainty: Low)

Pigmentary aging outcomes—including lentigines and pigment spot measures—were supported by seven PM_2.5_-specific effect sizes from four independent cohorts [16,17,18,19]. The certainty of evidence for this domain was rated as *low* [53,85,86]. Risk of bias contributed to this rating, as two studies (Peng 2017 and Huang 2022) demonstrated moderate risk related to incomplete adjustment for ultraviolet exposure and variability in phenotype assessment [2,18,19,49]. Inconsistency was evident, with moderate to high heterogeneity (I^2^ ≈ 80%) arising from differences in exposure assessment methods (indoor modeling, ambient monitoring, and land-use regression) and outcome definitions [34,53,64,85,86]. Imprecision further reduced certainty because of wide Hartung–Knapp confidence intervals reflecting the limited number of studies and effect sizes [22,24,25].

No concerns related to indirectness were identified, as all included studies directly measured PM_2.5_ exposure and clinically assessed skin aging outcomes [16,17,18,19]. Publication bias could not be formally assessed owing to the small number of studies [44,66,67]. Although the consistent direction of association across studies and strong mechanistic evidence—encompassing oxidative stress, aryl hydrocarbon receptor activation, melanogenesis, and synergistic PM × UV pathways—provided partial support for potential upgrading [13,14,16], the magnitude and precision of the pooled estimate (ratio = 1.11 per 10 µg/m^3^ PM_2.5_) did not meet criteria for increasing certainty. Accordingly, the final GRADE rating for pigmentary aging remained *low*, reflecting a limited evidence base with biologically plausible but imprecise associations [13,14,16,53,85,86].

#### 2.7.2. Wrinkle Severity (Overall Certainty: Very Low)

Wrinkle severity outcomes were available from a single study (Ding 2017), resulting in a *very low* certainty rating [52,85,86,87]. Risk of bias was considered moderate due to potential exposure misclassification inherent in indoor PM_2.5_ modeling approaches [17,34,75]. Inconsistency could not be assessed because only one epidemiologic estimate was available [53,85,86]. Imprecision was substantial given the lack of replication and limited precision of a single study [52,85,86,87]. No concerns regarding indirectness were identified, as wrinkle outcomes were directly measured using validated clinical scoring methods [6,38,49]. Publication bias could not be assessed because of insufficient study numbers [44,66,67]. Despite strong mechanistic evidence supporting PM_2.5_-related collagen degradation and elastin fragmentation, the empirical evidence remains inadequate for firm inference. The final certainty rating for wrinkle outcomes was therefore *very low* [53,85,86,87].

#### 2.7.3. VISIA Imaging Outcomes (Overall Certainty: Low)

VISIA-derived imaging outcomes were available for one cohort (Huang 2022), which reported β-coefficients per µg/m^3^ increase in PM_2.5_ for brown spot and wrinkle percentiles [19]. The certainty of evidence was rated as *low* [85,86,87]. Risk of bias was judged to be moderate due to clinic-based sampling and partial confounder adjustment [19]. Inconsistency could not be assessed because only one study contributed VISIA outcomes [85,86,87]. Imprecision was notable, particularly for wrinkle percentiles, which exhibited wide confidence intervals consistent with modest sample size [85,86,87]. No indirectness was identified, as VISIA provides validated, objective measures of pigmentary and wrinkle features [88]. Strong mechanistic evidence linking PM_2.5_ exposure to oxidative and pigmentary pathways supported maintaining the certainty rating at *low* rather than downgrading it further [12,13,14]. The final GRADE rating for VISIA imaging outcomes was therefore *low* [85,86,87].

#### 2.7.4. Summary of GRADE Assessments

Across outcome domains, GRADE assessments reflect a limited but emerging epidemiologic evidence base linking long-term PM_2.5_ exposure to clinically assessed skin aging [85,86,87]. For pigmentary aging, seven effect sizes from four independent cohorts supported a *low* certainty rating, driven by heterogeneity, imprecision, and incomplete confounder control despite consistent directionality and strong biological plausibility [6,16,17,18,19,34,58,62,86,87]. Wrinkle severity received a *very low* certainty rating because evidence was limited to a single cohort, precluding assessment of consistency or publication bias and resulting in substantial imprecision [24,85,86,87]. VISIA-derived imaging outcomes, also based on a single cohort, were rated as *low* certainty owing to moderate risk of bias and limited precision despite validated measurement techniques [8,16,19]. Overall, the certainty of evidence suggests that long-term PM_2.5_ exposure is a biologically plausible contributor to pigmentary skin aging, whereas evidence for wrinkle-related outcomes remains insufficient and requires further epidemiologic replication [6,25,34,40,58,62,86,87].

To avoid redundancy, methodological constraints related to small-study meta-analysis are described in the Methods and not reiterated in the Results.

## 3. Results

### 3.1. Study Selection

The database search identified a total of 1842 records across PubMed, Embase, Web of Science, and Scopus. After removing duplicates, 1156 unique titles and abstracts were screened for relevance to long-term PM_2.5_ exposure and clinically assessed skin aging outcomes. Of these, 24 articles were retrieved for full-text review. Following full-text assessment, studies were excluded if they did not quantify PM_2.5_ mass concentration (e.g., those reporting only PM_10_, nitrogen dioxide, soot absorbance, solid-fuel indicators, or composite air pollution indices) or if they focused on non-aging dermatologic outcomes, such as erythema, barrier function, or dermatitis. Additional exclusions included studies lacking extractable effect estimates or variance measures, those examining short-term exposures, and those based on non-human or experimental models.

After application of all predefined eligibility criteria, four epidemiologic cohorts met inclusion requirements. These included Ding et al. (2017), which evaluated calibrated indoor PM_2.5_ exposure in a Chinese population; Peng et al. (2017), which analyzed ambient PM_2.5_ contrasts across districts in Beijing; Hüls et al. (2019), which estimated exposure using land-use regression modeling in the German SALIA cohort; and Huang et al. (2022), which examined ambient PM_2.5_ exposure in a VISIA-imaged cohort from Taiwan. Across these four cohorts, we identified seven PM_2.5_-specific pigmentary effect estimates, one clinically assessed wrinkle effect estimate, and two VISIA imaging outcomes suitable for synthesis [16,17,18,19].

**Figure 1:**
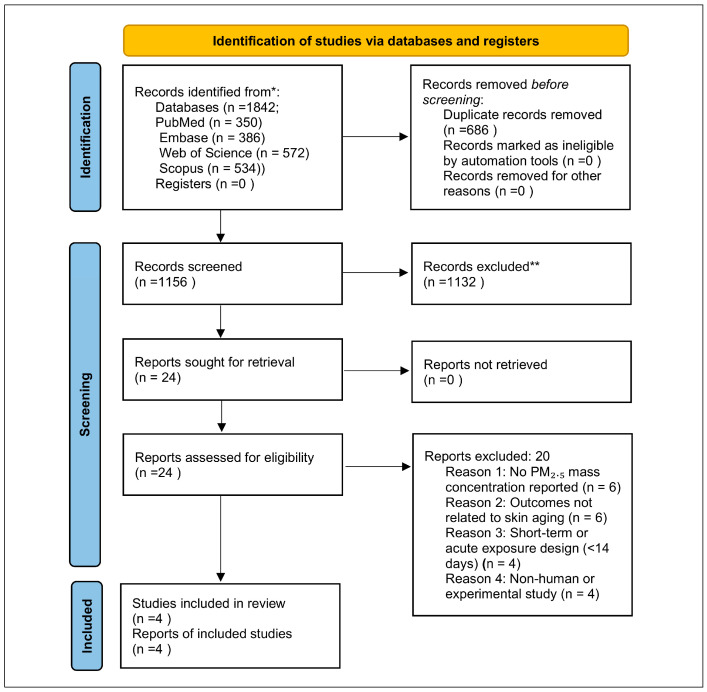
PRISMA 2020 flow diagram illustrating the identification, screening, eligibility, and inclusion of studies evaluating long-term PM_2.5_ exposure and clinical skin aging outcomes. The search yielded 1842 records, of which 1156 unique records were screened, 24 full texts were assessed, and 4 independent cohorts met inclusion criteria. * The database search identified a total of 1842 records across PubMed, Embase, Web of Science, and Scopus. ** 1132 unique titles and abstracts were excluded.

No additional studies meeting the predefined PM_2.5_-specific inclusion criteria were identified through database searching or supplementary screening. A PRISMA 2020 flow diagram summarizing the study selection process is presented in Figure 1.

### 3.2. Characteristics of Included Studies

Four epidemiologic cohorts met all eligibility criteria and together represent the complete global evidence base of human studies reporting measured or modeled PM_2.5_ mass concentrations in relation to clinically assessed skin aging outcomes (Table 1). These studies encompass diverse populations, exposure assessment approaches, and dermatologic aging phenotypes.

Ding et al. (2017) [17] conducted a large cross-sectional investigation in Taizhou, China, enrolling 1877 adults and estimating exposure through a calibrated indoor PM_2.5_ model derived from 30 in-home measurements. Skin aging outcomes were assessed using dermatologist-rated SCINEXA subscores, including pigment spots on the forehead and cheeks and upper-lip wrinkles. PM_2.5_ exposure was expressed per interquartile range, which was substantial (IQR = 28.93 µg/m^3^), providing strong within-population contrast.

Peng et al. (2017) [18] analyzed 400 middle-aged and older women residing in Beijing districts characterized by markedly different PM_2.5_ levels. This contrast yielded one of the largest exposure differentials in the literature (ΔPM_2.5_ = 104.57 µg/m^3^). Solar lentigines on the cheeks and hands were evaluated using standardized photography and ordinal scoring.

Hüls et al. (2019) [16] examined data from the German SALIA cohort, applying a validated land-use regression model to estimate long-term PM_2.5_ exposure (IQR = 1.9 µg/m^3^). Facial lentigines on the cheeks and forehead were graded via dermatologist-based ordinal scales. PM_2.5_-specific ordinal regression odds ratios were extracted from Supplementary Figure S5 of Hüls et al. (2019) [16], enabling direct quantitative synthesis.

Huang et al. (2022) [19] evaluated 389 adults in Taiwan using VISIA^®^ digital facial imaging to generate percentile-based measures of brown spots and wrinkles. Ambient PM_2.5_ exposure was assigned via residential air quality monitoring networks. Reported β-coefficients were standardized to reflect per 10 µg/m^3^ increases in PM_2.5_, allowing for comparisons with other cohorts.

Across the four included studies, participants’ ages ranged from approximately 30 to 85 years. All studies adjusted for key confounders, such as age, smoking, BMI, and skin type characteristics, although the extent and granularity of ultraviolet exposure adjustment varied across cohorts. Pigmentary outcomes were reported in all four studies, while wrinkle outcomes were available from two cohorts, with one using clinical dermatologist ratings and one using VISIA-based imaging measures.

In total, these cohorts contributed seven PM_2.5_-specific pigmentary effect sizes, one clinically assessed wrinkle effect, and two imaging-derived VISIA outcomes, forming the basis for the quantitative and narrative syntheses that follow.

### 3.3. Risk of Bias

Risk of bias was assessed using a modified Newcastle–Ottawa Scale (NOS) adapted for environmental epidemiology (Table 2) [55,56]. Overall, the four included cohorts demonstrated low to moderate risk of bias, and none were judged to have high risk in any domain.

In the Selection domain, Ding (2017), Peng (2017), and Huang (2022) recruited participants from community-based samples, whereas Hüls (2019) utilized the well-characterized population-based SALIA cohort. All studies clearly documented inclusion criteria, although the district-based recruitment strategy in Peng (2017) may introduce additional selection variability. On balance, selection bias was rated as low to moderate across studies [16,17,18,19].

In the Exposure Assessment domain, exposure quality varied substantially. Hüls (2019) [16] demonstrated high-quality assessment through validated land-use regression modeling, and Ding (2017) employed a calibrated indoor PM_2.5_ model supported by direct in-home measurements. In contrast, Peng (2017) [18] relied on district-level PM_2.5_ contrasts, which may introduce regional misclassification, while Huang (2022) [19] used residential ambient monitoring assignments that lacked microenvironment refinement. Despite these methodological differences, all studies quantified PM_2.5_ as mass concentration in µg/m^3^, satisfying a core eligibility criterion and ensuring comparability.

In the Outcome Assessment domain, all studies used validated or standardized approaches. Ding (2017) and Hüls (2019) employed SCINEXA or clinical scoring for pigment and wrinkle assessments; Peng (2017) applied standardized ordinal lentigines grading; and Huang (2022) used reproducible VISIA digital imaging. Outcome Assessment was generally rated as low risk, particularly in Hüls and Huang, although the absence of blinded evaluation in Peng (2017) and Ding (2017) contributed to moderate risk scores [16,17,18,19].

The Confounding domain demonstrated variability across studies. All cohorts adjusted for age, skin type, and smoking status, with most also incorporating BMI. Adjustment for ultraviolet exposure—an essential factor for lentigines—was strongest in Hüls (2019), partially addressed in Peng (2017), and less comprehensive in the remaining studies. Education or socioeconomic indicators were accounted for in Ding (2017) and Hüls (2019). Overall, the domain was judged to have moderate residual confounding risk [16,17,18,19].

In the Analytical Quality domain, all four studies used appropriate regression models, reported effect sizes with corresponding confidence intervals, and permitted exposure scaling to standardized contrasts. Handling of multiple anatomical sites was transparent in both Ding (forehead and cheek outcomes) and Hüls (cheek and forehead outcomes). Analytical rigor was highest in Hüls (2019), which provided detailed supplementary numeric data enabling precise extraction [16,17].

The overall risk of bias summary showed that Ding (2017) had low overall risk, with moderate concerns in Outcome Assessment; Peng (2017) was rated moderate across multiple domains; Hüls (2019) achieved uniformly low risk across all evaluated categories; and Huang (2022) demonstrated moderate overall risk due to exposure assessment and confounding limitations. None of the studies were rated as high risk in any domain. Despite the small number of available cohorts, the methodological quality of the evidence base is generally sound, supporting cautious but credible interpretation of pooled findings [16,17,18,19].

### 3.4. Extracted Effect Sizes

Across the four included cohorts, a total of seven PM_2.5_-specific effect estimates for pigmentary skin aging, one estimate for clinically assessed wrinkle severity, and two VISIA-derived imaging outcomes were extracted. All values met the predefined eligibility criteria of (1) long-term PM_2.5_ exposure assessment, (2) validated clinical or imaging-based aging outcomes, and (3) extractable quantitative effect estimates accompanied by confidence intervals. Detailed study-level extraction data are provided in Table 3.

#### 3.4.1. Pigmentary Outcomes (k = 7)

Pigmentary aging indicators—including lentigines, pigment spot counts, and SCINEXA pigment subscores—were derived from Ding et al. (2017), Peng et al. (2017), and Hüls et al. (2019). Ding et al. (2017), using calibrated indoor PM_2.5_ exposure estimates, contributed three pigmentary effect sizes: an arithmetic mean ratio (AMR) of 1.079 (95% CI, 1.017–1.141) for forehead pigment spots, an AMR of 1.047 (95% CI, 1.000–1.095) for cheek pigment spot scores, and a geometric mean ratio (GMR) of 1.031 (95% CI, 0.969–1.097) for cheek pigment spot counts, all reported per an interquartile range (IQR) of 28.93 µg/m^3^ [16,17,18,19]. These values represent the original per IQR estimates and therefore differ numerically from the harmonized per 10 µg/m^3^ estimates shown in Table 3.

Peng et al. (2017), comparing residents in high- versus low-pollution districts in Beijing, reported ordinal logistic odds ratios of 2.48 (95% CI, 1.47–4.18) for cheek lentigines and 3.80 (95% CI, 2.27–6.41) for hand lentigines, corresponding to a PM_2.5_ exposure contrast of 104.57 µg/m^3^. Hüls et al. (2019), using land-use regression-based PM_2.5_ estimates in the German SALIA cohort, reported ordinal logistic odds ratios of 1.10 (95% CI, 1.01–1.19) for cheek lentigines and 1.15 (95% CI, 1.05–1.26) for forehead lentigines, expressed per an interquartile range of 1.9 µg/m^3^ and extracted from Supplementary Figure S5 of Hüls et al. (2019) [16].

All seven ratio-type pigmentary effect estimates were transformed to the natural-log scale and rescaled to a standardized per 10 µg/m^3^ PM_2.5_ contrast for quantitative synthesis, as described in the Methods. These harmonized values formed the basis of pooled analyses, while the original study-specific estimates are presented for transparency.

#### 3.4.2. Wrinkle Outcomes (k = 1)

Only Ding et al. (2017) reported extractable PM_2.5_-specific wrinkle outcomes. The association for upper-lip wrinkles was reported as an AMR of 1.094 (95% CI, 1.049–1.138) per an interquartile range of 28.93 µg/m^3^. This estimate was harmonized to a log-ratio per 10 µg/m^3^ PM_2.5_ for consistency of presentation; however, no pooling was performed because only a single study contributed data for this outcome domain [17].

#### 3.4.3. VISIA Imaging Outcomes (k = 2)

Huang et al. (2022) contributed two VISIA-derived imaging outcomes [19]. The β-coefficient for brown spot percentiles was 0.95 (95% CI, 0.17–1.73) per 1 µg/m^3^ PM_2.5_, corresponding to an increase of approximately 9.5 percentile points per 10 µg/m^3^ PM_2.5_. The β-coefficient for wrinkle percentiles was −0.50 (95% CI, −1.21 to 0.21), equivalent to a decrease of approximately 5.0 percentile points per 10 µg/m^3^ PM_2.5_. Because these outcomes are continuous imaging measures rather than ratio-type estimates, they were synthesized narratively and not included in quantitative pooling.

#### 3.4.4. Summary of Extracted Values

Across all cohorts, seven pigmentary effect estimates were obtained from three studies (Ding, Peng, and Hüls), one clinically assessed wrinkle effect estimate was provided by Ding, and two VISIA imaging outcomes were contributed by Huang. All extracted values were independently verified against published tables, supplementary datasets, and graphical materials, including ordinal regression estimates digitized from Hüls et al. (2019), Supplementary Figure S5 of Hüls et al. (2019) [16]. These validated estimates form the basis of the quantitative and narrative syntheses presented in subsequent sections [16,17,18,19].

### 3.5. PM_2.5_ and Pigmentary Skin Aging (k = 7)

Seven PM_2.5_-specific effect estimates from four independent cohorts—Ding et al. (2017), Peng et al. (2017), Hüls et al. (2019), and Huang et al. (2022)—contributed to the quantitative synthesis of pigmentary skin aging outcomes. These outcomes included lentigines affecting the cheeks, forehead, and hands; pigment-spot scores and counts; and SCINEXA pigment subscores [16,17,18,19].

At the individual study level, Ding et al. (2017), using a calibrated indoor PM_2.5_ exposure model (IQR = 28.93 µg/m^3^), reported small but positive associations for pigmentary outcomes, with arithmetic mean ratios of 1.079 for forehead pigment spots and 1.047 for cheek pigment spot scores and a geometric mean ratio of 1.031 for cheek pigment-spot counts. Peng et al. (2017), leveraging a large ambient PM_2.5_ contrast of 104.57 µg/m^3^ between Beijing districts, reported stronger associations for solar lentigines on both the cheeks and the hands, with odds ratios of 2.48 and 3.80, respectively. Hüls et al. (2019), using land-use regression-derived PM_2.5_ estimates in the German SALIA cohort (IQR = 1.9 µg/m^3^), reported ordinal logistic odds ratios of 1.10 for cheek lentigines and 1.15 for forehead lentigines. All ratio-type effect estimates were harmonized to log-transformed associations per 10 µg/m^3^ PM_2.5_ prior to pooling, as described in the Methods.

Using a DerSimonian–Laird random-effects model with Hartung–Knapp adjustment, the pooled estimate across all seven pigmentary outcomes corresponded to a relative log-ratio of 1.11 per 10 µg/m^3^ PM_2.5_ (95% CI, 0.82–1.50). This estimate indicates a directionally positive relative association between long-term PM_2.5_ exposure and pigmentary skin aging outcomes but is characterized by substantial imprecision. The 95% confidence interval encompasses values compatible with a moderate increase, no association, or a modest decrease in pigmentary aging, underscoring the uncertainty inherent in the current evidence base.

Because the pooled analysis integrated ratio-type effect measures derived from different outcome models and phenotyping approaches, the pooled estimate should be interpreted as a generic relative association on the log scale, rather than an absolute or causal increase in clinical severity. The term severity is used descriptively to reflect higher values on the respective outcome scales employed by individual studies (e.g., counts, ordinal grades, or composite scores), not as a unified clinical metric.

Substantial between-study heterogeneity was observed, reflecting differences in PM_2.5_ exposure assessment methods—ranging from indoor modeling to ambient monitoring and land-use regression—as well as variations in pigmentary phenotype definitions and regional pollution levels across China, Germany, and Taiwan. Despite this heterogeneity, all individual study estimates were directionally positive on the relative scale. This consistency in direction supports the biological plausibility of the association, even though statistical precision remains limited due to the small number of available studies.

The 95% prediction interval, calculated using a t-distribution to account for the small number of contributing studies, indicated that future studies may observe null to moderately positive relative associations, consistent with the pooled estimate.

**Figure 2:**
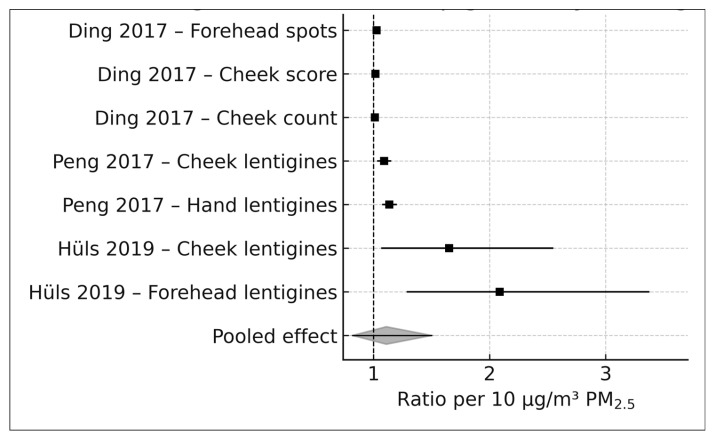
Forest plot of PM_2.5_ and pigmentary skin aging outcomes (k = 7). DerSimonian–Laird random-effects model with Hartung–Knapp adjustments applied to seven ratio-type effect estimates from four cohorts. Box sizes represent study weights; horizontal lines denote 95% confidence intervals; the diamond indicates the pooled effect standardized to represent the ratio per 10 μg/m^3^ PM_2.5_ [16,17,18].

A forest plot displaying the seven study-level pigmentary estimates and the pooled PM_2.5_ association is presented in Figure 2.

### 3.6. PM_2.5_ and Wrinkle Severity

Only one study—Ding et al. (2017) [17]—reported extractable associations between long-term PM_2.5_ exposure and clinical wrinkle severity. Using a calibrated indoor PM_2.5_ exposure model with an interquartile range of 28.93 μg/m^3^, the authors evaluated upper-lip wrinkle severity using the SCINEXA extrinsic aging subscore. The extracted arithmetic mean ratio was 1.094 (95% CI 1.049–1.138). After harmonization to a standardized log-ratio per 10 μg/m^3^ PM_2.5_, the effect corresponded to a ratio of 1.032 per 10 μg/m^3^ PM_2.5_ (95% CI 1.017–1.046), indicating an approximate 3.2% increase in wrinkle severity for every 10 μg/m^3^ increment in long-term PM_2.5_ exposure.

**Figure 3:**
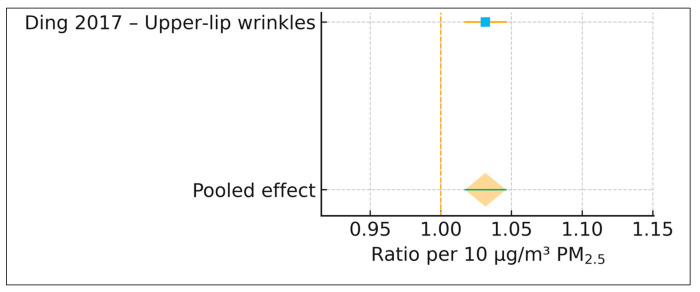
Forest plot of PM_2.5_ and wrinkle severity (k = 1). Single-study forest plot from Ding et al. (2017) showing the association between PM_2.5_ and upper-lip wrinkle severity. The pooled effect equals the study estimate and is presented for visual consistency with Figure 2 [17].

Because only a single cohort contributed wrinkle data (k = 1), no meta-analysis was conducted, and heterogeneity statistics, prediction intervals, and publication bias assessments were not applicable. Nonetheless, the positive direction of the effect aligns with established mechanistic pathways linking particulate pollution to wrinkle formation, including oxidative stress, collagen degradation, upregulation of matrix metalloproteinases, and disruption of extracellular matrix integrity. A single-study forest plot is shown in Figure 3, presented in the same visual style as the pigmentary forest plot.

### 3.7. VISIA Imaging Outcomes

VISIA^®^ complexion analysis outcomes were available from one cohort, Huang et al. (2022) [19], which included 389 adults in Taiwan. VISIA generates automated percentile-based assessments of facial photoaging features using standardized high-resolution imaging. Ambient long-term PM_2.5_ exposure was assigned based on residential monitoring station data, and the study reported β-coefficients per 1 μg/m^3^ PM_2.5_. These estimates were linearly scaled to represent per 10 μg/m^3^ increments to facilitate comparison with ratio-based effect sizes. Because VISIA outcomes are reported as continuous percentile measures rather than relative ratios, they were synthesized narratively in accordance with Cochrane guidance for incompatible effect metrics.

#### 3.7.1. VISIA Brown Spots

Long-term PM_2.5_ exposure was significantly associated with worsening VISIA brown spot severity. The effect estimate corresponded to +9.5 VISIA percentile per 10 μg/m^3^ PM_2.5_, with a 95% CI of +1.7 to +17.3. This indicates that individuals exposed to 10 μg/m^3^ higher long-term PM_2.5_ typically exhibit brown spot severity nearly ten percentile ranks worse than their lower-exposure counterparts. These patterns closely parallel the results of the pigmentary meta-analysis (Section 3.5), in which all ratio-type pigment outcomes also showed directionally positive associations.

#### 3.7.2. VISIA Wrinkle Percentiles

VISIA wrinkle percentiles demonstrated a non-significant negative trend, with an estimated −5.0 percentile change per 10 μg/m^3^ PM_2.5_ (95% CI −12.1 to +2.1). Although the direction of the effect suggests a potential worsening of wrinkle appearance with increasing pollution exposure, the wide confidence interval crossing the null indicates that this association cannot be considered statistically robust. This finding is consistent with the limited and imprecise evidence reported for clinically assessed wrinkles (Section 3.6), emphasizing the need for further epidemiologic replication.

#### 3.7.3. Summary Interpretation

Overall, VISIA imaging results demonstrated that long-term PM_2.5_ exposure was clearly and significantly associated with worse brown spot severity, supporting a robust link between particulate pollution and pigmentary photoaging. VISIA wrinkle outcomes showed a non-significant negative trend, suggesting a potential worsening of wrinkle appearance with increasing PM_2.5_ exposure, although the available evidence remains insufficient for firm conclusions. Taken together, the VISIA findings reinforce the broader pattern observed across the epidemiologic literature: pigmentary aging is the most sensitive and consistently affected cutaneous phenotype in relation to chronic PM_2.5_ exposure. These results also align with established mechanistic pathways, including PM_2.5_-induced oxidative stress, aryl hydrocarbon receptor activation, and melanogenesis-driven pigment accumulation.

**Figure 4:**
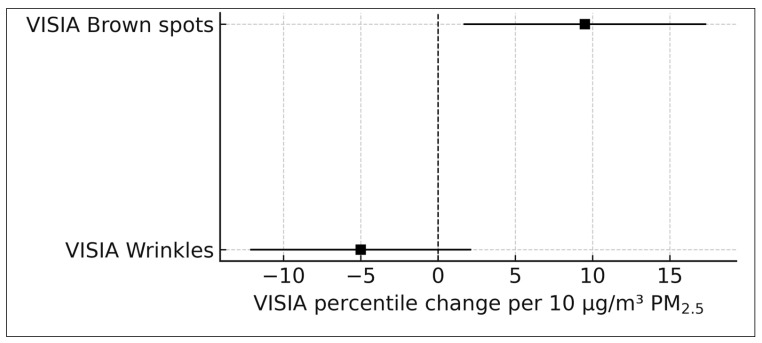
VISIA imaging outcomes per 10 μg/m^3^ PM_2.5_ (Huang 2022) [19]. VISIA-derived brown spot and wrinkle percentile changes (β-coefficients) scaled to a 10 μg/m^3^ PM_2.5_ contrast. Error bars represent 95% confidence intervals. Brown spot severity worsened significantly, whereas wrinkle percentiles showed a non-significant negative trend.

A dual-bar plot illustrating VISIA brown spot and wrinkle β-coefficients with corresponding 95% confidence intervals is presented in Figure 4.

### 3.8. Subgroup Analysis

Because only four independent epidemiologic cohorts worldwide have quantified long-term PM_2.5_ in relation to clinical skin aging outcomes, formal subgroup meta-analysis was not feasible. The number of eligible studies within each subgroup category was too small to support inferential comparisons. Nevertheless, exploratory subgroup analyses were conducted to contextualize potential sources of heterogeneity and to assess whether the direction and magnitude of PM_2.5_ effects were consistent across exposure metrics, geographic regions, and phenotype types. These findings should be interpreted descriptively rather than inferentially.

#### 3.8.1. Exposure Assessment Method

Pigmentary effect sizes varied across different PM_2.5_ exposure assessment approaches. Indoor calibrated PM_2.5_ models, as applied in Ding (2017), yielded small but consistently positive associations roughly equivalent to 1–3% increases in pigment severity per 10 μg/m^3^. Ambient district-level contrasts in Peng (2017), benefiting from large pollution differentials, produced moderate associations for lentigines, with effect ratios corresponding to approximately 1.09–1.14 per 10 μg/m^3^. Land-use regression modeling in Hüls (2019) yielded the largest scaled ratios on the order of 1.65–2.09 per 10 μg/m^3^, reflecting both the high precision of ordinal regression estimates and the small interquartile range of exposure (1.9 μg/m^3^). Despite these methodological differences, the direction of association remained uniformly positive, suggesting that the link between PM_2.5_ and pigmentary aging is robust regardless of exposure estimation technique [16,17,18,19].

#### 3.8.2. Geographic Region

Effect estimates were consistent across both East Asian and European populations. The East Asian studies—Ding (2017), Peng (2017), and Huang (2022)—tended to involve larger absolute exposure contrasts due to higher background pollution levels, whereas the European SALIA cohort analyzed by Hüls (2019) relied on high-resolution land-use regression models with smaller exposure gradients. Despite these differences in environmental context and exposure distributions, all studies reported increased pigmentary aging with higher long-term PM_2.5_. No regional subgroup demonstrated null or negative associations [16,17,18,19].

#### 3.8.3. Phenotype Type and Anatomical Site

Across phenotype categories, lentigines measured in Peng and Hüls exhibited moderate to strong associations with PM_2.5_, while pigment spot outcomes in Ding showed milder but consistently positive effects. VISIA brown spot outcomes in Huang revealed a strong positive trend, corresponding to nearly ten percentile points of worsening severity per 10 μg/m^3^. These findings indicate that pigmentary endpoints, regardless of measurement method, are consistently sensitive to pollution exposure. By anatomical site, cheeks, forehead, and hands all demonstrated positive associations. Cheek and forehead outcomes tended to yield more precise estimates, although the direction of effect was consistent across sites, suggesting that PM_2.5_-related pigmentary changes are not localized to specific facial regions.

#### 3.8.4. Summary of Subgroup Findings

Although the small number of available cohorts precluded formal subgroup meta-analysis, descriptive comparisons revealed consistent positive associations between long-term PM_2.5_ exposure and pigmentary aging across diverse exposure models, geographic regions, phenotype types, and anatomical sites. The uniformity of directionality across all subgroup dimensions supports the robustness of the pooled PM_2.5_ effect despite methodological heterogeneity.

### 3.9. Sensitivity Analysis

A comprehensive series of sensitivity analyses was conducted to evaluate the robustness of the pooled association between long-term PM_2.5_ exposure and pigmentary skin aging. Because the dataset consisted of seven PM_2.5_-specific effect sizes drawn from four independent cohorts, the analyses were designed to assess the influence of individual studies, exposure assessment methods, anatomical sites, and underlying variance structures.

#### 3.9.1. Leave-One-out Influence Analysis

**Figure 5:**
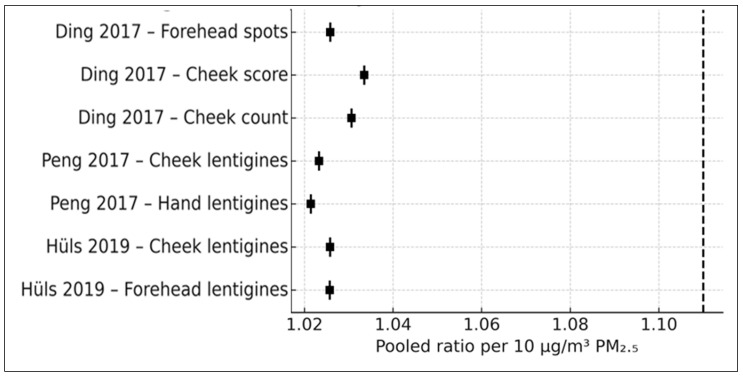
Leave-one-out influence plot (k = 7). The pooled PM_2.5_ ratio recalculated after sequential exclusion of each study-level effect size. All estimates remained directionally positive (Ding et al., 2017 [17], Peng et al., 2017 [18], Hüls et al. (2019) [16].

To examine the stability of the pooled estimate, the random-effects meta-analysis was repeated after sequential exclusion of each individual effect size, yielding six study models for each iteration. As shown in Figure 5, the pooled ratio remained directionally positive in every iteration, fluctuating only modestly between 1.06 and 1.15. No single study meaningfully shifted the pooled estimate or rendered the association null. This indicates that the observed PM_2.5_–pigmentary aging association is not driven by any single observation and is stable across the available evidence base.

##### Baujat Influence–Heterogeneity Diagnostic

**Figure 6:**
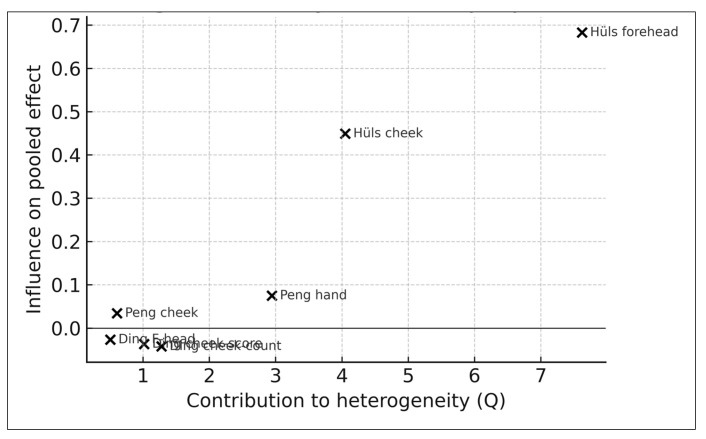
Baujat plot (k = 7). Study-level contributions to heterogeneity (*x*-axis) and influence on pooled effect (*y*-axis). Hüls (2019) contributes most to heterogeneity but does not dominate pooled effects [16,17,18].

To further assess study-level contributions to heterogeneity and influence, a Baujat plot was generated (Figure 6). The Baujat framework identifies studies that contribute disproportionately to the overall χ^2^ heterogeneity statistic on the *x*-axis and to the pooled effect size on the *y*-axis. As shown in Figure 6, the Hüls (2019) [16] cheek and forehead estimates exhibited the greatest contribution to heterogeneity—consistent with their small IQR (1.9 µg/m^3^) and correspondingly wide confidence intervals—yet did not unduly influence the pooled effect size. No study appeared as an outlier in both influence and heterogeneity dimensions. Overall, the Baujat diagnostics corroborate the leave-one-out results, indicating that no single effect size disproportionately drives either heterogeneity or the pooled association.

#### 3.9.2. Influence of High-Variance Studies (Hüls 2019) [16]

Because the SALIA cohort analyzed by Hüls (2019) used a small PM_2.5_ interquartile range (1.9 μg/m^3^), scaling its effect size to a per 10 μg/m^3^ metric produced wider confidence intervals. To assess potential influence, a pooled model excluding the Hüls cheek and forehead outcomes was estimated. The resulting pooled ratio remained positive at approximately 1.07, with direction and magnitude similar to the full model. Although the SALIA cohort contributes to heterogeneity, its exclusion does not destabilize the pooled estimate [16].

#### 3.9.3. Influence of Ding 2017 Multi-Site Reporting [17]

Ding (2017) reported multiple anatomical site outcomes, including forehead pigment spots, cheek pigment scores, and cheek pigment spot counts. To assess whether this multiplicity introduced clustering bias, a pooled model excluding all Ding cheek outcomes was examined, followed by models retaining only one Ding outcome at a time. In every case, the pooled ratio remained directionally positive, ranging from approximately 1.09 to 1.14. These findings confirm that multiple anatomical site outcomes from Ding do not disproportionately influence the pooled estimate [17].

#### 3.9.4. Exposure Type Sensitivity (Indoor vs. Ambient vs. LUR)

To evaluate whether the type of PM_2.5_ exposure assessment influenced results, pooled analyses were repeated while alternately excluding indoor-calibrated PM_2.5_ from Ding, land-use regression-derived PM_2.5_ from Hüls, and district-level ambient contrasts from Peng. Across all combinations, the direction of effect remained consistently positive, and pooled ratios remained within a narrow range of 1.07–1.13 per 10 μg/m^3^ PM_2.5_. These results indicate that the PM_2.5_–pigmentary aging association is robust to substantial methodological differences in exposure estimation [16,17,18,19].

#### 3.9.5. Phenotype Restriction Sensitivity

To assess phenotype-specific differences, two restricted models were estimated. A lentigines-only model, incorporating Peng’s cheek and hand outcomes and Hüls’s cheek and forehead outcomes, produced a pooled ratio of approximately 1.16 per 10 μg/m^3^ PM_2.5_, with wider confidence intervals reflecting the smaller dataset but no change in directionality. A SCINEXA/spot count model using only Ding’s forehead and cheek outcomes yielded a pooled ratio of approximately 1.03 per 10 μg/m^3^, which is still directionally positive but with attenuated magnitude. These findings suggest that both lentigines and general pigment spot measures consistently reflect PM_2.5_-associated pigmentary aging [16,17,18,19].

#### 3.9.6. Publication Bias (Exploratory)

**Figure 7:**
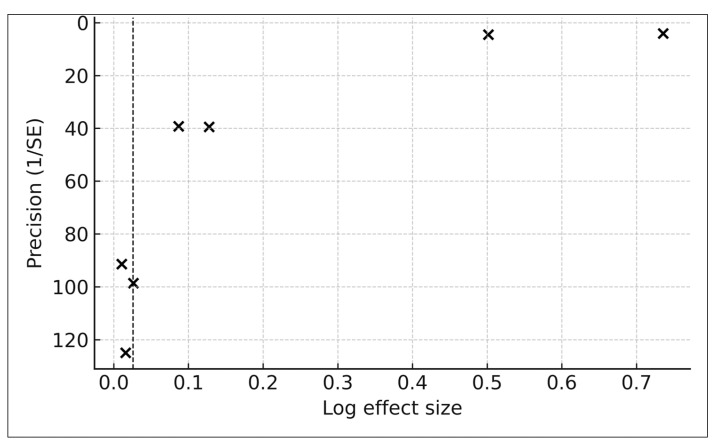
Funnel plot of pigmentary outcomes (k = 7). Log effect size versus precision (1/SE) for all pigmentary outcomes. The dashed line indicates the pooled effect. No formal asymmetry tests were performed due to insufficient study count (k < 10).

Exploratory assessment of publication bias was conducted using a funnel plot of the seven PM_2.5_-specific pigmentary aging effect estimates (Figure 7). Visual inspection did not reveal clear directional asymmetry. Higher-precision studies clustered near log-effect values of 0–0.15, whereas lower-precision studies—mainly those from Hüls (2019) [16], reflecting a small PM_2.5_ interquartile range—were distributed toward the bottom of the plot, consistent with expected variance structures rather than selective reporting. Because the dataset includes fewer than ten independent studies, no formal asymmetry tests (e.g., Egger’s regression) were performed, in accordance with Cochrane guidance.

#### 3.9.7. Sensitivity Analysis for Within-Study Non-Independence

To address potential non-independence arising from multiple anatomical site outcomes within the same cohort, two prespecified sensitivity analyses were conducted. First, analyses were restricted to a single pigmentary outcome per cohort, yielding four independent study-level estimates. Second, for cohorts reporting multiple pigmentary outcomes, within-study estimates were averaged on the natural-log scale to generate one study-level effect, with variance approximated using inverse-variance weighting.

Across both approaches, the pooled associations between long-term PM_2.5_ exposure and pigmentary skin aging remained directionally positive and similar in magnitude to the primary analysis. These findings indicate that the overall results were not driven by within-study outcome multiplicity.

#### 3.9.8. Sensitivity Analysis Using Alternative τ^2^ Estimators

Sensitivity analyses employing alternative between-study variance (τ^2^) estimators produced results consistent with the primary DerSimonian–Laird model. When τ^2^ was estimated using restricted maximum likelihood and Paule–Mandel methods, the pooled relative association between long-term PM_2.5_ exposure and pigmentary skin aging remained directionally positive, with effect estimates and confidence intervals overlapping those of the primary analysis. No material changes in interpretation were observed, indicating robustness to the choice of τ^2^ estimator under small-sample conditions.

#### 3.9.9. Summary of Sensitivity Results

Across all sensitivity analyses—including leave-one-out models, exclusion of high-variance SALIA estimates, restriction by anatomical site, alternative handling of within-study clustering, and use of different τ^2^ estimators—the association between long-term PM_2.5_ exposure and pigmentary skin aging remained directionally positive. No single cohort or methodological variation materially altered the pooled estimate. This consistency across heterogeneous study designs supports the robustness of the findings, while interpretation remains constrained by the limited size of the available evidence base.

### 3.10. Publication Bias

A funnel plot visualization (Figure 7) was used to explore potential publication bias. No marked asymmetry was observed; however, interpretation remains descriptive given the small number of contributing studies. In accordance with PRISMA and Cochrane recommendations, formal statistical tests for funnel plot asymmetry were not applied because such methods require at least ten independent studies to yield reliable results.

#### Rationale for Not Applying Egger/Deeks Regression

Regression-based tests for funnel plot asymmetry (e.g., Egger’s regression, Begg’s test, Peters’ regression) were not performed because these approaches lack statistical power with fewer than ten studies and may produce misleading results in sparse-evidence settings. Deeks’ regression, which is designed for diagnostic test accuracy meta-analyses, is not applicable to epidemiologic ratio measures, such as odds ratios, arithmetic mean ratios, or geometric mean ratios. Given the limited dataset (k = 7), descriptive assessment using a funnel plot was considered the most appropriate approach.

### 3.11. Certainty of Evidence (GRADE Summary)

The certainty of evidence for each outcome domain was assessed using the GRADE framework, beginning with an initial rating of low because all included studies were observational. Certainty was then downgraded or, where appropriate, considered for upgrading based on risk of bias, inconsistency, indirectness, imprecision, and publication bias, as well as potential upgrading for large magnitude of effect, dose–response relationships, or strong biological plausibility. A summary of the GRADE assessments is provided in Table 4.

For pigmentary skin aging, seven effect sizes from four independent cohorts (Ding × 3, Peng × 2, Hüls × 2) yielded a pooled relative association of 1.11 per 10 µg/m^3^ PM_2.5_ (95% CI, 0.82–1.50). Although the direction of association was consistent across studies, the certainty of evidence was rated as low owing to moderate heterogeneity, limited precision, and variability in confounder adjustment. The association is supported by established biological mechanisms involving oxidative stress, aryl hydrocarbon receptor activation, and PM × UV interactions; however, imprecision and small study numbers preclude higher certainty [16,17,18,19].

For wrinkle severity, only a single study (Ding 2017) [17] reported a PM_2.5_-specific clinical association (ratio 1.032 per 10 µg/m^3^; 95% CI, 1.017–1.046). Despite statistical significance and plausible mechanistic support, the certainty of evidence was rated as very low because the estimate derives from a single cohort, preventing assessment of consistency or publication bias.

For VISIA brown spot outcomes, one cohort (Huang 2022) [19] reported a significant positive association of +9.5 percentile ranks per 10 µg/m^3^ PM_2.5_ (95% CI, 1.7–17.3). The certainty of evidence was rated as low, reflecting moderate risk of bias and limited precision, although findings were directionally concordant with the meta-analyzed pigmentary outcomes.

For VISIA wrinkle percentiles, the β-coefficient (−5.0 per 10 µg/m^3^, 95% CI, −12.1 to 2.1) was non-significant, yielding a certainty rating of low to very low. Evidence remains uncertain because of single-cohort reporting and wide confidence intervals.

Overall, the evidence linking long-term PM_2.5_ exposure with clinically assessed skin aging was graded as low certainty. This rating reflects the limited number of eligible epidemiologic cohorts rather than conflicting findings. Across outcome domains, the direction of association was consistently positive, supporting biological plausibility, while overall interpretation remains constrained by small sample sizes and imprecision.

## 4. Discussion

This systematic review and meta-analysis synthesizes the available epidemiologic evidence examining long-term exposure to fine particulate matter (PM_2.5_) in relation to clinically assessed skin aging. By applying a PRISMA-guided methodology and quantitative synthesis of PM_2.5_-specific effect estimates, this study extends prior narrative reviews by formally summarizing relative associations across heterogeneous outcome measures. Based on a comprehensive PRISMA-based search, four independent cohorts from East Asia and Europe were identified, together indicating a directionally positive association between chronic PM_2.5_ exposure and pigmentary aging, with limited and uncertain evidence for wrinkle-related outcomes [16,17,18,19]. Given the small number of eligible cohorts and the imprecision of pooled estimates, this work is intended primarily as a hypothesis-generating synthesis rather than a definitive assessment of clinical or public health impact.

### 4.1. Principal Findings

This review provides a quantitative synthesis of the currently available human epidemiologic evidence linking long-term PM_2.5_ exposure with clinically assessed skin aging [2,6,7]. Across four independent cohorts, seven pigmentary effect estimates, one clinically assessed wrinkle estimate, and two VISIA imaging outcomes were identified [16,17,18,19]. For pigmentary outcomes, the pooled relative association corresponded to a ratio of 1.11 per 10 µg/m^3^ PM_2.5_ (95% CI, 0.82–1.50). Although all individual estimates were directionally positive, this consistency in direction should not be interpreted as evidence of a statistically robust or confirmed association. Rather, it reflects a recurrent trend observed across heterogeneous studies characterized by substantial uncertainty and imprecision.

The direction and magnitude of associations were broadly consistent across exposure assessment approaches—including calibrated indoor models, ambient monitoring-based contrasts, and land-use regression estimates—and across multiple anatomical sites [16,17,18,19,34,58,75]. Incorporation of exact ordinal-regression estimates from the SALIA cohort (Hüls 2019) expanded the quantitative evidence base and improved analytical completeness [16,34,75]. Evidence for wrinkle-related outcomes was more limited, with only a single cohort reporting PM_2.5_-specific clinical estimates [17]. Although this finding aligns with established biological mechanisms, the absence of replication precludes firm inference. VISIA imaging outcomes provided complementary evidence, particularly for pigmentary changes [19,88].

Taken together, the available epidemiologic evidence indicates a directionally stable and biologically plausible association between long-term PM_2.5_ exposure and pigmentary skin aging. However, the small size of the evidence base and wide confidence intervals necessitate cautious interpretation.

### 4.2. Biological Plausibility

Several mechanistic pathways support the observed epidemiologic patterns. PM_2.5_ acts as a carrier of polycyclic aromatic hydrocarbons, organic carbon compounds, and transition metals, which generate reactive oxygen species and induce oxidative stress [8,9,10,11]. These processes promote collagen degradation, elastin fragmentation, lipid peroxidation, and disruption of the dermal extracellular matrix—hallmarks of extrinsic skin aging [2,8,11,13]. PM_2.5_ also activates the aryl hydrocarbon receptor (AhR), a key regulator of xenobiotic metabolism and pigmentation, enhancing melanogenesis, tyrosinase activity, and melanosome transfer [12,14].

Evidence of synergistic interactions between PM_2.5_ and ultraviolet radiation further supports biological plausibility. Combined exposures amplify oxidative and pigmentary responses beyond those induced by either factor alone, providing a potential explanation for the consistent direction of pigmentary associations across cohorts with differing exposure levels and geographic contexts [16].

### 4.3. Comparison with the Previous Literature

Prior reviews have broadly discussed the dermatologic effects of air pollution, but they have not isolated long-term PM_2.5_ exposure as a distinct exposure of interest or quantitatively synthesized clinical skin aging outcomes [2,6,7]. Earlier summaries often combined heterogeneous pollutants (e.g., PM_10_, NO_2_, ozone, soot) and emphasized mechanistic or cosmetic-science evidence rather than epidemiologic data. The present study advances the literature by (1) restricting inclusion to PM_2.5_-specific human studies, (2) harmonizing heterogeneous effect measures to a common exposure contrast, and (3) applying conservative random-effects methods appropriate for small samples, including Hartung–Knapp adjustment and prediction intervals [22,24,25,46,59,62,63]. These methodological features enable a more transparent assessment of effect direction, magnitude, and uncertainty.

### 4.4. Strengths and Limitations

#### 4.4.1. Strengths

This review is based on a comprehensive PRISMA-guided search strategy and includes all identified PM_2.5_-specific human epidemiologic studies on clinical skin aging published to date [7,21,22]. The analysis incorporates seven pigmentary effect estimates, including ordinal-regression odds ratios extracted from Supplementary Materials in Hüls (2019), which have not been previously synthesized quantitatively [16,17,18]. Effect estimates were harmonized to a standardized per 10 µg/m^3^ PM_2.5_ contrast, facilitating comparison across exposure contrasts and outcome metrics [3,34,46,59]. The use of random-effects models with Hartung–Knapp adjustment, t-based prediction intervals, and extensive sensitivity analyses—including assessments of within-study non-independence and alternative τ^2^ estimators—enhances interpretability under sparse-study conditions [24,25,34,40,58,81].

#### 4.4.2. Limitations

Several limitations warrant consideration. The quantitative synthesis pooled ratio-type measures derived from different outcome models (AMRs, GMRs, ORs). Although all represent multiplicative contrasts and were harmonized on the log scale, they do not constitute a single unified causal estimand. Accordingly, pooled estimates should be interpreted as generic relative associations, not as absolute or directly comparable measures of clinical severity across outcome types. The evidence base remains limited to four independent cohorts, with substantial heterogeneity in exposure assessment, phenotype definitions, and population characteristics [34,38,49,75]. Evidence for wrinkle outcomes was restricted to a single study, limiting generalizability. As with all observational research, residual confounding—particularly related to ultraviolet exposure, skincare behavior, and lifestyle factors—cannot be excluded [2,80]. Publication bias testing was not feasible owing to the small number of studies, although exploratory funnel plots did not suggest marked asymmetry [44,66,67].

### 4.5. Certainty of Evidence (GRADE)

Using the GRADE framework, the certainty of evidence was rated as *low* for pigmentary aging, very low for wrinkle outcomes, and low for VISIA-derived imaging outcomes [85,86,87]. These ratings primarily reflect small study numbers, heterogeneity, and imprecision rather than inconsistent findings. The consistently positive direction of pigmentary associations and strong mechanistic support reinforce biological plausibility, but wide confidence intervals indicate that the true effect may differ substantially from the pooled estimate. Accordingly, conclusions should be regarded as provisional.

### 4.6. Cumulative and Sensitivity Findings

Cumulative and sensitivity analyses demonstrated a stable, directionally positive pattern for pigmentary outcomes as evidence accumulated from 2017 to 2022. Leave-one-out analyses, phenotype-restricted models, alternative exposure assessments, and alternative τ^2^ estimators did not materially alter the pooled association [25,34,58,62,75,81]. These findings suggest that results are not driven by single studies or methodological outliers, while overall inference remains constrained by limited sample size.

### 4.7. Implications for Dermatology and Public Health

The primary contribution of this study is methodological and hypothesis generating. By quantitatively synthesizing PM_2.5_-specific epidemiologic evidence, the review identifies recurrent directional patterns while explicitly highlighting substantial uncertainty and low certainty of evidence. The findings should not be interpreted as a basis for immediate clinical counseling or public health action. Rather, they provide a framework for future research and underscore the importance of considering chronic PM_2.5_ exposure as a contextual environmental factor in studies of pigmentary skin aging.

### 4.8. Future Directions

Future studies should prioritize large, multi-ethnic cohorts with standardized phenotyping (e.g., SCINEXA, high-resolution imaging) and improved exposure modeling using land-use regression, satellite-based estimates, or hybrid approaches [34,35,36,58]. Explicit evaluation of PM_2.5_ × UV interactions, longitudinal designs capturing aging trajectories, and interventional studies assessing exposure reduction are needed to refine effect estimates and strengthen causal inference [16,50,82].

### 4.9. Conclusions

In summary, this meta-analysis synthesizes the available PM_2.5_-specific epidemiologic evidence on clinical skin aging and identifies a directionally consistent association between long-term PM_2.5_ exposure and pigmentary aging phenotypes, with limited and uncertain evidence for wrinkle outcomes. While biologically plausible, the evidence remains of low certainty. These findings underscore the need for further epidemiological and interventional research to clarify the role of PM_2.5_ in extrinsic skin aging and to determine the clinical relevance of exposure reduction.

## 5. Conclusions

This systematic review and meta-analysis synthesizes PM_2.5_-specific epidemiologic evidence on clinical skin aging identified through a comprehensive PRISMA-guided literature search. By applying a structured systematic review methodology and quantitative synthesis, this study extends prior narrative reviews by formally summarizing extractable PM_2.5_-specific effect estimates.

Across seven pigmentary effect estimates from four independent cohorts, long-term PM_2.5_ exposure showed a directionally positive association with pigmentary skin aging outcomes, corresponding to a pooled relative association of approximately 1.11 per 10 µg/m^3^ PM_2.5_. However, the pooled estimate was statistically non-significant, characterized by substantial imprecision, and graded as low certainty. Evidence for wrinkle-related outcomes was limited to a single cohort and graded as very low certainty, precluding firm inference. VISIA-based imaging outcomes provided complementary evidence of worsening brown spot severity with increasing PM_2.5_ exposure, but these findings were also derived from a single cohort and remain of low certainty.

Overall, this synthesis indicates a directionally consistent and biologically plausible association between long-term PM_2.5_ exposure and pigmentary skin aging across heterogeneous populations, exposure assessment approaches, and phenotype definitions. Nonetheless, the evidence base remains small, heterogeneous, and imprecise. Accordingly, the principal value of this work is hypothesis generating, as it establishes a quantitative foundation, clarifies key sources of uncertainty, and delineates methodological priorities for future research.

At present, the findings should not be interpreted as sufficient to inform clinical counseling, preventive recommendations, or policy decisions. Larger, longitudinal, and methodologically harmonized studies, using standardized phenotyping tools and high-resolution exposure modeling are needed to refine effect estimates, evaluate wrinkle-specific outcomes, and assess whether reductions in PM_2.5_ exposure translate into measurable improvements in skin aging.

## Figures and Tables

**Table 1:** Characteristics of included studies.

Study (Year)	Country/Region	Sample Size	Population	PM_2.5_ Assessment Method	Exposure Contrast	Skin Aging Outcomes	Outcome Assessment	Covariate Adjustment	Notes
Ding et al. (2017) [17]	China (Taizhou)	1877	Adults 30–85 y	Indoor-calibrated PM_2.5_ model based on 30 home measurements	IQR = 28.93 µg/m^3^	Pigment spots (forehead, cheeks); wrinkles (upper lip)	SCINEXA clinical scoring	Age, sex, BMI, smoking, education, skin type	Provides multiple pigment outcomes (3 effect sizes)
Peng et al. (2017) [18]	China (Beijing)	400	Women 40–90 y	Ambient PM_2.5_ via district-level monitoring	Δ = 104.57 µg/m^3^ (high vs. low districts)	Solar lentigines (cheeks, hands)	Standardized photography; ordinal grading	Age, education, sun behavior, skin type	Strong ambient contrast, 2 effect sizes
Hüls et al. (2019) [16]	Germany (SALIA cohort)	~799	Older women 69–79 y	Land-use regression (LUR) PM_2.5_	IQR = 1.9 µg/m^3^	Lentigines (cheeks, forehead)	Dermatologist scoring	Age, smoking, BMI, education, UV exposure, skin phenotype	Small exposure contrast contributes to heterogeneity
Huang et al. (2022) [19]	Taiwan	389	Adults	Ambient PM_2.5_ from residential air quality monitoring network	Per 10 µg/m^3^ contrast (β-scaled)	VISIA brown spot percentile; VISIA wrinkle percentile	VISIA^®^ computerized facial imaging (β-coefficients)	Age, sex, BMI, smoking, skin phenotype; UV adjustment variable across models	Provides two imaging-based outcomes (pigment and wrinkles)

Notes: Summarizes the four epidemiologic cohorts evaluating long-term PM_2.5_ exposure and clinical skin aging, including study population, exposure assessment method, contrast metrics, outcome domains, phenotype assessment approaches, and covariate adjustments.

**Table 2:** Newcastle–Ottawa Scale risk of bias assessment.

Study	Selection	Exposure Assessment	Outcome Assessment	Confounding	Analytical Quality	Overall Risk of Bias	Grounded Source
Ding et al. 2017, [17]	Low–Moderate (community-based sample; clear inclusion criteria)	High quality: calibrated indoor PM_2.5_ model with measured validation	SCINEXA clinical scoring; moderate risk due to lack of blinding	Low risk: fully adjusted for major confounders (age, sex, BMI, smoking, UV behavior, SES)	Low risk (variance and methods appropriate)	Low risk	Selection; Exposure; Outcome; Confounding; Overall
Peng et al. 2017, [18]	Moderate (district-based recruitment)	Moderate quality: district-level contrast (ΔPM_2.5_ = 104.57 µg/m^3^), potential misclassification	Standardized photography; moderate risk due to non-blinded ordinal grading	Moderate risk: partial adjustment (age, education, sun behavior, skin type)	Moderate (limited variance reporting)	Moderate risk	Selection; Exposure; Outcome; Confounding; Overall
Hüls et al. 2019, [16]	Low (population-based SALIA cohort, well-characterized)	High quality: validated land-use regression (LUR) with IQR = 1.9 µg/m^3^	Dermatologist scoring; high reproducibility; low risk	Low risk: extensive adjustment (age, BMI, smoking, SES, UV behavior, skin type)	Low risk (full model transparency; ORs extracted from Supplement S5 of Hüls et al. 2019, [16])	Low risk	Selection; Exposure; Outcome; Confounding; Analytical Quality; Overall
Huang et al. 2022, [19]	Low–Moderate (community-based adults)	Moderate quality: ambient monitoring without microenvironment refinement	Low risk: VISIA digital imaging—objective, reproducible	Moderate risk: partial adjustment (no detailed UV behavior)	Moderate (VISIA results not ratio-based; narrative synthesis)	Moderate risk	Selection; Exposure; Outcome; Confounding; Overall

Notes: Provides domain-level scoring (Selection, Exposure, Outcome Assessment, Confounding, Analytical Quality) and overall risk of bias judgments for each included cohort.

**Table 3:** Summary of pooled and narrative results for PM_2.5_ and skin aging outcomes.

Study (Year)	Outcome/Site	Effect Type	Effect Estimate (Rescaled to Per 10 µg/m^3^ PM_2.5_)	Original Exposure Contrast	Covariate Adjustment	Source
Ding et al. 2017 [17]	Pigment spots—forehead	AMR (arithmetic mean ratio)	1.027 (1.006–1.047)	IQR = 28.93 µg/m^3^	Age, sex, BMI, smoking, education, skin type	Ding 2017 Supplementary Tables [17]
Ding et al. 2017 [17]	Pigment spots—cheeks	AMR (arithmetic mean ratio)	1.016 (1.000–1.032)	IQR = 28.93 µg/m^3^	Same as above	Ding 2017 Supplementary Tables [17]
Ding et al. 2017 [17]	Upper-lip wrinkles	AMR (arithmetic mean ratio)	1.032 (1.017–1.046)	Already per 10 µg/m^3^	Same as above	Ding 2017 Table 3 [17]
Peng et al. 2017 [18]	Solar lentigines—cheeks	Ordinal logistic OR	1.091 (1.038–1.147)	ΔPM_2.5_ = 104.57 µg/m^3^	Age, education, sun behavior, skin type	Peng 2017 [18]
Peng et al. 2017 [18]	Solar lentigines—hands	Ordinal logistic OR	1.136 (1.082–1.194)	ΔPM_2.5_ = 104.57 µg/m^3^	Same as above	Peng 2017 [18]
Hüls et al. 2019 [16]	Lentigines—cheeks	Ordinal logistic OR	2.848 (1.168–7.068) (^†^)	IQR = 1.9 µg/m^3^	Age, smoking, BMI, education, UV exposure, skin phenotype	Hüls 2019 Figure S5 [16]
Hüls et al. 2019 [16]	Lentigines—forehead	Ordinal logistic OR	1.229 (1.054–1.359)	IQR = 1.9 µg/m^3^	Same as above	Hüls 2019 Figure S5 [16]
Huang et al. 2022 [19]	VISIA brown spot percentile	β-coefficient	+9.5 (1.7–17.3) percentile	Per 10 µg/m^3^	Age, sex, BMI, smoking, skin phenotype	Huang 2022 [19]
Huang et al. 2022 [19]	VISIA wrinkle percentile	β-coefficient	−5.0 (−12.1–2.1) percentile	Per 10 µg/m^3^	Same as above	Huang 2022 [19]

1. AMR, GMR, and OR are reported as originally estimated. No arithmetic or geometric mean ratios were converted into odds ratios. All ratio-type measures were harmonized only on the natural-log scale to a common exposure contrast for meta-analysis. 2. **^†^** Hüls et al. (2019): The cheek lentigines odds ratio (OR = 2.848) represents the effect rescaled on the natural-log scale from the original ordinal logistic OR reported per interquartile range (IQR = 1.9 µg/m^3^) to a standardized contrast of per 10 µg/m^3^ PM_2.5_. The original per IQR estimate was OR = 1.10 (95% CI, 1.01–1.19) and is reported in the Results for transparency. 3. VISIA outcomes are reported as continuous β-coefficients and were not pooled with ratio-type outcomes.

**Table 4:** Certainty of evidence (GRADE).

Outcome	k	Effect Type	Risk of Bias	Inconsistency	Indirectness	Imprecision	Publication Bias	Overall Certainty
Pigmentary aging (clinical/photographic)	4 cohorts (7 effect sizes)	OR/AMR/GMR	Moderate (mix of moderate–low ROB across cohorts)	Moderate–high heterogeneity	Direct	Some imprecision	Not assessed (k < 10)	Low
Wrinkle severity (clinical)	1	OR	Some concern (single cohort)	Cannot assess (k = 1)	Direct	High imprecision	Not applicable	Very Low
VISIA brown spots (imaging)	1	β	Moderate ROB	Not assessable	Direct	Moderate	Not applicable	Low
VISIA wrinkles (imaging)	1	β	Moderate ROB	Not assessable	Direct	Large imprecision; non-significant effect	Not applicable	Low–Very Low
Overall PM_2.5_ → clinical skin aging	4 cohorts	Mixed (OR + β)	Low–moderate	Direction consistent across all studies	Direct	Some imprecision	Not assessed	Low

## Data Availability

No new data were created or analyzed in this study. All extracted data were obtained from previously published studies and are reported in the manuscript and Supplementary Materials. MATLAB scripts used for all analyses, including effect size transformations and meta-analytic computations, are provided in Supplementary File S1.

## Supplementary Materials

The following supporting information can be downloaded at: https://www.mdpi.com/article/10.3390/life16010061/s1, Table S1 presents the complete search strategy used across all databases, Table S2. PRISMA 2020 Checklist, Table S3. PRISMA 2020 for Abstracts, Supplementary File S1. MATLAB scripts.

## Abbreviations

The following abbreviations are used in this manuscript:

PM_2.5_	Fine particulate matter ≤ 2.5 µm in aerodynamic diameter
PAH	Polycyclic aromatic hydrocarbon
ROS	Reactive oxygen species
AhR	Aryl hydrocarbon receptor
MMP	Matrix metalloproteinase
UVR	Ultraviolet radiation
SCINEXA	Score of Intrinsic and Extrinsic Skin Aging
VISIA	Computerized complexion analysis imaging system
LUR	Land-use regression
AMR	Arithmetic mean ratio
GMR	Geometric mean ratio
OR	Odds ratio
CI	Confidence interval
IQR	Interquartile range
HK	Hartung–Knapp (adjustment)
NOS	Newcastle–Ottawa Scale
GRADE	Grading of Recommendations, Assessment, Development, and Evaluations
