# Supplementary material for: Long-Term PM2.5 Exposure and Clinical Skin Aging: A Systematic Review and Meta-Analysis of Pigmentary and Wrinkle Outcomes
Source: Life (Basel). 2025 Dec 30;16(1):61. doi: 10.3390/life16010061 (PMC12843363; doi:10.3390/life16010061)
Supplement: Supplementary file 1 [file life-16-00061-s001.zip › life-4031905 supplementaryS1-S3.pdf]

Table S1. presents the complete search strategy used across all databases, including fully enumerated Boolean search strings for PubMed, Embase, Web of Science, and Scopus. Both controlled-vocabulary terms and free-text keywords related to $PM_{2.5}$ exposure, photoaging, lentigines, pigment spots, wrinkles, and VISIA outcomes are included.

**Database**	**Search String**	**Limits**	**Date Range**
PubMed	("PM2.5"[Mesh] OR "PM2.5" OR "PM 2.5" OR "fine particulate matter" OR "particulate matter" OR "air pollution" OR "ambient pollution" OR "airborne particles") AND ("Skin Aging"[Mesh] OR "skin aging" OR "photoaging" OR "photo-aging" OR "pigment" OR "pigmentary" OR "lentigines" OR "solar lentigo" OR "age spots" OR "wrinkle" OR "wrinkles" OR "dermal aging" OR "cutaneous aging" OR "VISIA" OR "complexion analysis")	None	Inception–18 Nov 2025
Embase	('pm2.5'/exp OR 'pm2.5' OR 'pm 2.5' OR 'fine particulate matter' OR 'particulate matter'/exp OR 'air pollution'/exp) AND ('skin aging'/exp OR 'skin ageing' OR 'photoaging'/exp OR 'pigmentation'/exp OR 'lentigo'/exp OR 'age spot' OR 'wrinkle'/exp OR 'wrinkles' OR 'dermal ageing' OR 'visia' OR 'skin imaging')	None	Inception–18 Nov 2025
Web of Science	TS = (("PM2.5" OR "PM 2.5" OR "fine particulate matter" OR "air pollution" OR "particulate matter") AND ("skin aging" OR "skin ageing" OR "photoaging" OR "pigment*" OR "lentigines" OR "solar lentigo" OR "age spots" OR "wrinkle*" OR "VISIA"))	None	Inception–18 Nov 2025
Scopus	(TITLE-ABS-KEY("PM2.5" OR "PM 2.5" OR "fine particulate matter" OR "particulate matter" OR "air pollution") AND TITLE-ABS-KEY("skin aging" OR "photoaging" OR "pigment" OR "pigmentary" OR "lentigines" OR "solar lentigo" OR "wrinkle" OR "wrinkles" OR "VISIA"))	None	Inception–18 Nov 2025

Table S2. PRISMA 2020 Checklist.

Section / Topic	Item #	Checklist Item (Exact Wording)	Location in Manuscript
Title	1	Identify the report as a systematic review.	Title page
Abstract	2	See the PRISMA 2020 for Abstracts checklist.	Structured Abstract
Rationale	3	Describe the rationale for the review in the context of existing knowledge.	Introduction, paragraphs 1–4
Objectives	4	Provide an explicit statement of the objective(s) or question(s) the review addresses.	Introduction, final paragraph
Eligibility criteria	5	Specify the inclusion and exclusion criteria for the review and how studies were grouped for the syntheses.	Sections 2.1.–2.2.
Information sources	6	Specify all databases, registers, websites, organisations, reference lists and other sources searched or consulted to identify studies. Specify the date when each source was last searched or consulted.	Section 2.1-2.2
Search strategy	7	Present the full search strategies for all databases, registers and websites, including any filters and limits used.	Supplementary Table S1
Selection process	8	Specify the methods used to decide whether a study met the inclusion criteria of the review, including how many reviewers screened each record and each report retrieved, whether they worked independently, and if applicable, details of automation tools used in the process.	Section 2.1-2.2

Data collection process	9	Specify the methods used to collect data from reports, including how many reviewers collected data from each report, whether they worked independently, any processes for obtaining or confirming data from study investigators, and if applicable, details of automation tools used in the process.	Section 2.3.1
Data items (10a)	10a	List and define all outcomes for which data were sought. Specify whether all results that were compatible with each outcome domain in each study were sought (e.g. for all measures, time points, analyses), and if not, the methods used to decide which results to collect.	Section 2.3.1
Data items (10b)	10b	List and define all other variables for which data were sought (e.g. participant and intervention characteristics, funding sources). Describe any assumptions made about any missing or unclear information.	Section 2.3.1
Study risk of bias assessment	11	Specify the methods used to assess risk of bias in the included studies, including details of the tool(s) used, how many reviewers assessed each study and whether they worked independently, and if applicable, details of automation tools used in the process.	Section 2.3.2
Effect measures	12	Specify for each outcome the effect measure(s) (e.g. risk ratio, mean difference) used in the synthesis or presentation of results.	Section 2.4.1
Synthesis methods (13a)	13a	Describe the processes used to decide which studies were eligible for each synthesis (e.g. tabulating the study intervention characteristics and comparing against the planned groups for each synthesis (item #5)).	Section 2.6.1
Synthesis methods (13b)	13b	Describe any methods required to prepare the data for presentation or synthesis, such as handling of missing summary statistics, or data conversions.	Section 2.4.1
Synthesis methods (13c)	13c	Describe any methods used to tabulate or visually display results of individual studies and syntheses.	Section 2.6.6 + Figures
Synthesis methods (13d)	13d	Describe any methods used to synthesize results and provide a rationale for the choice(s). If meta-analysis was performed, describe the model(s), method(s) to identify the presence and extent of statistical heterogeneity, and software package(s) used.	Sections 2.4.2–2.4.4
Synthesis methods (13e)	13e	Describe any methods used to explore possible causes of heterogeneity among study results (e.g. subgroup analysis, meta-regression).	Sections 2.7.1 and 3.8
Synthesis methods (13f)	13f	Describe any sensitivity analyses conducted to assess robustness of the synthesized results.	Sections 2.7.2 and 3.9
Reporting bias assessment	14	Describe any methods used to assess risk of bias due to missing results in a synthesis (arising from reporting biases).	Sections 2.7.3 and 3.10
Certainty assessment	15	Describe any methods used to assess certainty (or confidence) in the body of evidence for an outcome.	Section 2.8
Study selection (16a)	16a	Describe the results of the search and selection process, from the number of records identified in the search to the number of studies included in the review, ideally using a flow diagram.	Section 3.1 + Figure 1
Study selection (16b)	16b	Cite studies that might appear to meet the inclusion criteria, but which were excluded, and explain why they were excluded.	Section 3.1
Study characteristics	17	Cite each included study and present its characteristics.	Section 3.2 + Table 1
Risk of bias in studies	18	Present assessments of risk of bias for each included study.	Section 3.3 + Table 2
Results of individual studies	19	For all outcomes, present, for each study: (a) summary statistics for each group (where appropriate) and (b) an effect estimate and its precision (e.g. confidence/credible interval), ideally using structured tables or plots.	Section 3.4 + Table 3
Results of syntheses (20a)	20a	For each synthesis, briefly summarise the characteristics and risk of bias among contributing studies.	Section 3.5
Results of syntheses (20b)	20b	Present results of all statistical syntheses conducted. If meta-analysis was done, present for each the summary estimate and its precision (e.g. confidence/credible interval) and measures of statistical heterogeneity. If comparing groups, describe the direction of the effect.	Section 3.5 + Figure 2
Results of syntheses (20c)	20c	Present results of all investigations of possible causes of heterogeneity among study results.	Section 3.8

Results of syntheses (20d)	20d	Present results of all sensitivity analyses conducted to assess the robustness of the synthesized results.	Section 3.9
Reporting biases	21	Present assessments of risk of bias due to missing results (arising from reporting biases) for each synthesis assessed.	Section 3.10
Certainty of evidence	22	Present assessments of certainty (or confidence) in the body of evidence for each outcome assessed.	Section 3.11 + Table 4
Discussion (23a)	23a	Provide a general interpretation of the results in the context of other evidence.	Section 4.1
Discussion (23b)	23b	Discuss any limitations of the evidence included in the review.	Section 4.4
Discussion (23c)	23c	Discuss any limitations of the review processes used.	Section 4.4
Discussion (23d)	23d	Discuss implications of the results for practice, policy, and future research.	Sections 4.7–4.9
Registration and protocol (24a)	24a	Provide registration information for the review, including register name and registration number, or state that the review was not registered.	Abstract; Methods (Section 2).
Registration and protocol (24b)	24b	Indicate where the review protocol can be accessed, or state that a protocol was not prepared.	Methods (Section 2).
Registration and protocol (24c)	24c	Describe and explain any amendments to information provided at registration or in the protocol.	None
Support	25	Describe sources of financial or non-financial support for the review, and the role of the funders or sponsors in the review.	Funding statement
Competing interests	26	Declare any competing interests of review authors.	Conflicts of Interest
Availability of data, code and other materials	27	Report which of the following are publicly available and where they can be found: template data collection forms; data extracted from included studies; data used for all analyses; analytic code; any other materials used in the review.	Data Availability Statement

Table S3. PRISMA 2020 for Abstracts – Completed Checklist.

Section	Item #	Checklist Item	Location in Abstract
Title	1	Identify the report as a systematic review.	Abstract title line
Background	2	Provide an explicit statement of the main objective(s) or question(s) the review addresses.	Abstract – Background/Objective
Methods – Eligibility criteria	3	Specify the inclusion and exclusion criteria for the review.	Abstract – Methods
Methods – Information sources	4	Specify the information sources (e.g. databases, registers) used to identify studies and the date when each was last searched.	Abstract – Methods
Methods – Risk of bias	5	Specify the methods used to assess risk of bias in the included studies.	Abstract – Methods
Methods – Synthesis	6	Specify the methods used to present and synthesise results.	Abstract – Methods
Results – Included studies	7	Indicate the number of studies and participants included in the review.	Abstract – Results
Results – Synthesis of results	8	Present results for main outcomes, preferably indicating the number of included studies and participants for each.	Abstract – Results
Discussion	9	Provide a brief summary of the limitations of the evidence included in the review.	Abstract – Conclusions
Interpretation	10	Provide a general interpretation of the results and important implications.	Abstract – Conclusions
Funding	11	Specify the primary source of funding for the review.	Abstract – Funding statement
Registration	12	Provide the register name and registration number, or state that the review was not registered.	Abstract – Registration
